# A Zebrafish Galectin-1 Isoform Is Expressed in Skin and Gills and Binds to Bacteria, Bacterial Adhesin Receptors, and Epidermal Mucus Glycans

**DOI:** 10.3390/ijms27093827

**Published:** 2026-04-25

**Authors:** Chiguang Feng, Kelsey Abernathy, Sheng Wang, Guanghui Zong, Nilli Zmora, Allison Shupp, Muddassar Iqbal, Lai-Xi Wang, Gerardo R. Vasta

**Affiliations:** 1 Department of Microbiology and Immunology, University of Maryland School of Medicine, UMB, and Institute of Marine and Environmental Technology, Baltimore, MD 21202, USA; 2 State Key Laboratory for Biocontrol, School of Life Sciences, Sun Yat-sen University, Guangzhou 510275, China; 3 Department of Chemistry and Biochemistry, University of Maryland, College Park, MD 20742, USA; 4 Department of Marine Biotechnology, Institute of Marine and Environmental Technology, University of Maryland Baltimore County, Baltimore, MD 21202, USA; 5 Northeastern University, Boston, MA 02115, USA

**Keywords:** zebrafish galectin, epidermal mucus, adhesin receptors, microbial glycans, mucus glycans, antibacterial defense

## Abstract

Galectins are a functionally diverse family of β-galactosyl-binding lectins that are ubiquitously present in animal species, with key roles in development and immune regulation. Recently, galectins have been found to recognize microbial glycosylated moieties, but the detailed mechanisms of their innate immune functions in mucosal epithelia have remained elusive. The zebrafish (*Danio rerio*) represents an ideal genetically tractable model to address these questions, as the skin, gills, and gut display mucosal surfaces exposed to the environment. In this study, we investigated the range of endogenous and microbial glycans that are recognized by zebrafish galectin Drgal1 present in epidermal mucus, which would be consistent with defense functions against a bacterial challenge. Results revealed that zebrafish galectin isoform Drgal1-L2 can recognize selected bacterial glycans, as well as zebrafish mucus glycans and cell-surface receptors for bacterial adhesins such as fibronectin (K_D_ = 1.593 × 10^−6^ M) and CD147 (K_D_ = 1.115 × 10^−6^ M). Furthermore, preliminary experiments revealed that Drgal1-L2 may hinder bacterial adhesion to epidermal mucus in about 50% at 2.5 μg/mL. Our results suggest that Drgal1-L2 present in epidermal mucus can prevent access of pathogenic bacteria to the epithelial cell surface by alternate or synergic binding to bacterial glycans and to zebrafish mucus components and epithelial receptors for bacterial adhesins. Thus, the present study provides key information for the testing of the abovementioned hypothesis by implementing gene-silencing approaches targeting both zebrafish Drgal1-L2 and its ligands.

## 1. Introduction

Galectins are a structurally conserved family of β-galactosyl-binding lectins with a unique sequence motif in their carbohydrate recognition domains (CRDs) [1,2]. Based on the organization of their polypeptide subunits, galectins are classified into three types: proto, chimera, and tandem repeat galectins [3]. Galectins are expressed in the cytoplasm of most cell types and can be secreted into the extracellular space, where they can bind to soluble glycoconjugates, as well as to those associated to the cell membrane and the extracellular matrix [1,2,4]. Through binding and cross-linking with endogenous carbohydrate ligands, galectins are involved in various biological functions, such as early development, tissue repair, and immune regulation, and play critical roles in cancer, obesity, inflammation, and diabetes [1,5,6,7,8]. Recent studies, however, have revealed that galectins can also bind to exogenous carbohydrate ligands such as glycans on the surfaces of viruses, bacteria, and eukaryotic parasites and function as pattern recognition receptors in innate immune responses [9,10,11]. In addition to their well-established functions as recognition factors, the roles of galectins as effector factors in innate immunity against microbial challenge have recently been characterized [10,11,12]. These include, for example, the direct killing of recognized bacteria [11,12,13,14,15] and opsonization followed by phagocytosis and intracellular killing of potential pathogens [16,17]. Moreover, the “subversion” of the recognition and effector functions of galectins from the host by viral and bacterial pathogens and eukaryotic parasites along their co-evolutionary process has been documented [18,19,20].

The zebrafish (*Danio rerio*) has become an ideal genetically tractable model for studying innate and adaptive immune responses to infectious challenge [21,22]. In previous research aimed at establishing the zebrafish as a model for molecular and structural/functional studies of galectins, we characterized the molecular diversity, ontogenic expression, and temporo-spatial localization of the zebrafish galectin repertoire [23]. Our initial functional studies focused on the roles of zebrafish prototype galectin (Drgal1) in skeletal and heart muscle development, as well as retinal photoreceptor regeneration, by recognition of endogenous cell-surface carbohydrate ligands displayed by these tissues [24,25,26]. More recently, our studies revealed that Drgal1-L2 can recognize the envelope glycoprotein of infectious hematopoietic necrosis virus (IHNV) and inhibit viral attachment to epithelial cells [27]. Further, we determined the crystal structure of Drgal1-L2 in complex with N-acetyllactosamine (LacNAc; Galβ1,4GlcNAc) and modeled the inhibitory effects of Drgal1-L2 on the viral envelope glycoprotein spikes that hinder IHNV attachment to the epithelial cell surface [28]. Importantly, our results revealed that Drgal1-L2 can also bind to glycans on the fish epithelial cell surface [27].

Based on these observations, we hypothesized that Drgal1 present in epidermal mucus can hinder the attachment of potentially pathogenic bacteria by binding to glycosylated adhesin receptors on the fish epithelium to prevent infection. As a first step in testing this hypothesis, this study was aimed at exploring the range of endogenous and exogenous microbial glycans recognized by extracellular Drgal1 that could be consistent with a defense function at the interface of the animal with its external environment. For this, we first characterized, in detail, the molecular features of Drgal1 isoforms and their tissue-specific expression and localization, with particular emphasis on fish mucosal tissues that are relevant to its potential immune function, such as skin, gills and gut, as well as epidermal mucus. Secondly, we analyzed the carbohydrate specificity of Drgal1-L2 by solid-phase assays and a glycan microarray, the potential binding specificity and affinity for the endogenous extracellular matrix, as well as cell-surface glycans by solid-phase assays and surface plasmon resonance analysis. Finally, we investigated the potential binding of Drgal1-L2 to exogenous ligands—namely, glycans from the bacterial surface—using a microbial microarray and the properties of its binding to whole bacteria and epidermal mucus that could support functions as a soluble defense molecule. Results revealed that Drgal1-L2 can bind to fish epithelial cell-surface glycoproteins such as fibronectin and CD147 that have been shown to function as receptors for bacterial adhesins, as well as both bacterial and epidermal mucus glycans.

## 2. Results

### 2.1. The Zebrafish Prototype Galectin Drgal1 Is Abundant in Skin, Gills, and Epidermal Mucus

**Figure 1:**
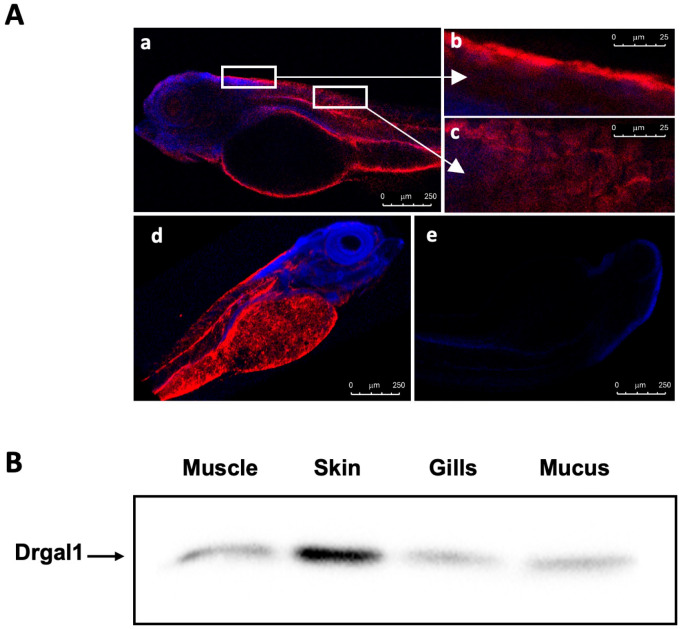
Localization of Drgal1 protein in zebrafish tissues and epidermal mucus. (**A**) Immunofluorescence detection of the Drgal1 protein in zebrafish larvae by confocal microscopy: Five days post fertilization (dpf), zebrafish larvae were fixed with 4% PFA, and whole-mount staining with anti-Drgal1 antibody was carried out to determine the localization of Drgal1 protein. The staining was analyzed and captured by confocal microscopy (Leica SP8 TCS). A representative staining of the sections from 5 larva of 3 independent experiments is shown (**a**). The boxed areas in (**a**) are magnified and shown in (**b**,**c**). A 3D structure was constructed with serial Z-stacks with 4.5 um distance, and the outside view (**d**) is shown. As a specificity control, zebrafish larvae were treated with pre-immune immunoglobulins (**e**). (**B**). Detection of Drgal1 in selected tissues and epidermal mucus of adult zebrafish by Western blot. Muscle, skin, gills, and epidermal mucus were extracted from adult wildtype zebrafish, and Western blotting was carried out with anti-Drgal1 antibody as described in the Materials and Methods. Representative images from a pool of 5 fishes of 3 independent pools are shown.

We used whole-mount immunostaining with the specific anti-Drgal1 to examine the Drgal1 distribution in zebrafish larvae. The results revealed that Drgal1 is present in several tissues (Figure 1(Aa))—most prominently, in the skin (Figure 1(Ab)), as well as the intestine and notochord (Figure 1(Ac)). While the image in (b) suggests that Drgal1 is localized in skin tissue, the more diffuse distribution devoid of DAPi staining, as evident from different planes, indicates that Drgal1 is extensively distributed throughout the external surface of the fish, most likely in the epidermal mucus (Figure 1(Ad)). Immunoglobulins purified from pre-immune serum (Day 0 of immunization course) showed no staining (Figure 1(Ae)). Assessment of the presence of Drgal1 in selected tissues and epidermal mucus of adult zebrafish by Western blot (Figure 1B) with the anti-Drgal1 antibody revealed that the Drgal1 protein (14.5 kDa) is localized in muscle, skin, gills, and epidermal mucus, confirming the findings in zebrafish larvae (Figure 1A), with the protein most abundant in skin. Consistent with a previous report [23], Drgal1 staining was also observed in the notochord.

### 2.2. Zebrafish Prototype Galectin Drgal1 Comprises Three Isoforms

**Figure 2:**
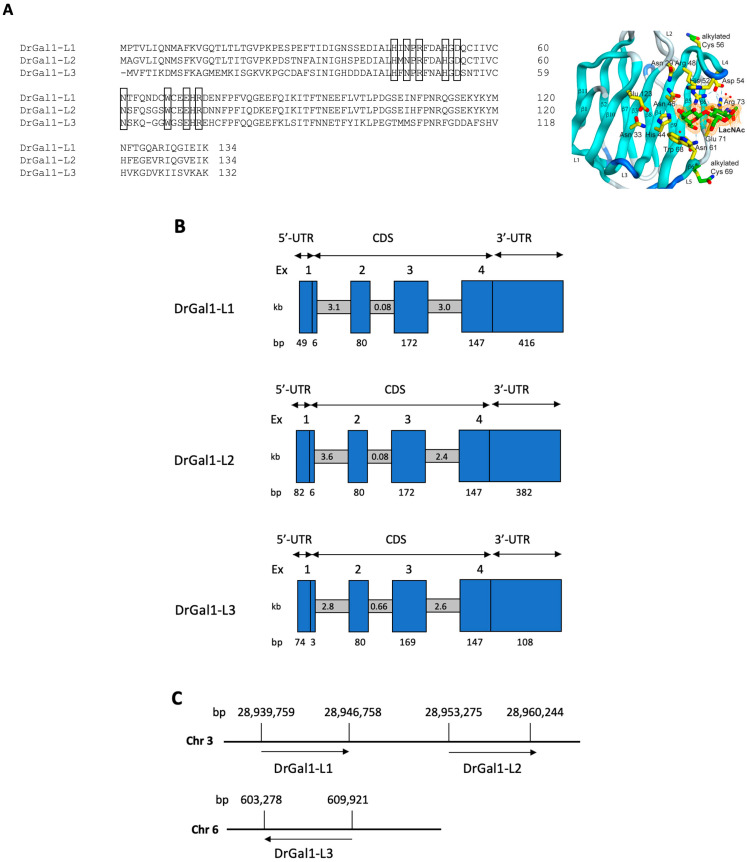
Isoforms of zebrafish Drgal1. (**A**) Comparison of the Drgal1 isoforms’ amino acid sequences: Amino acid sequences of Drgal1-L1, -L2, and L3 are aligned by the ClustalW program (https://www.ebi.ac.uk/Tools/msa/clustalo/, accessed on 13 November 2025). The amino acids that interact with LacNAc as determined from the 3D structure of the bovine galectin-1 [29] are boxed and are illustrated on the right panel, showing the Drgal1 CRD in complex with LacNAc, as published by Ghosh et al., 2019 [28]. (**B**) Gene organization of zebrafish galectin-1 isoforms Drgal1-L1, Drgal1-L2, and Drgal1-L3. The blue boxes represent exons, which are numbered at the top. The size of each exon (in bp) is indicated at the bottom. The grey horizontal boxes represent introns; their sizes are indicated in kb. (**C**) Chromosomal location and gene organization of zebrafish galectin-1 isoforms: schematic representation of galectins’ locations in chromosomes 3 (NCBI Reference Sequence: NC_007114.7) and 6 (NCBI Reference Sequence: NC_007117.7).

From the gene and transcript sequence information available in GenBank for the zebrafish (*Danio rerio*), three isoforms of Drgal1 can be identified: Drgal1-L1, -L2, and -L3 [23] (Figure 2A). A ClustalW alignment (https://www.ebi.ac.uk/Tools/msa/clustalo/) accesed on 10 February 2025) of the isoforms’ deduced protein sequences enabled confirmation of the presence of all key amino acid residues (shown in boxes) that interact with the LacNAc disaccharide ligand, as determined from the crystal structure of bovine galectin-1 [29]. Using these sequences, the gene organization, including the exon/intron structure, were determined for each Drgal1 isoform (Figure 2B). The three Drgal1 isoforms display very similar gene structures, composed of four exons separated by three introns, with all corresponding regions of similar size among the three isoforms and exons 1 and 2 being shorter than exons 3 and 4 in all three isoforms (Figure 2B). Genes encoding isoforms Drgal1-L1 and -L2 are located on chromosome 3, while the Drgal1-L3 isoform is located on chromosome 6 (Figure 2C).

### 2.3. Phylogenetic Analysis of the Drgal1 Isoforms

**Figure 3:**
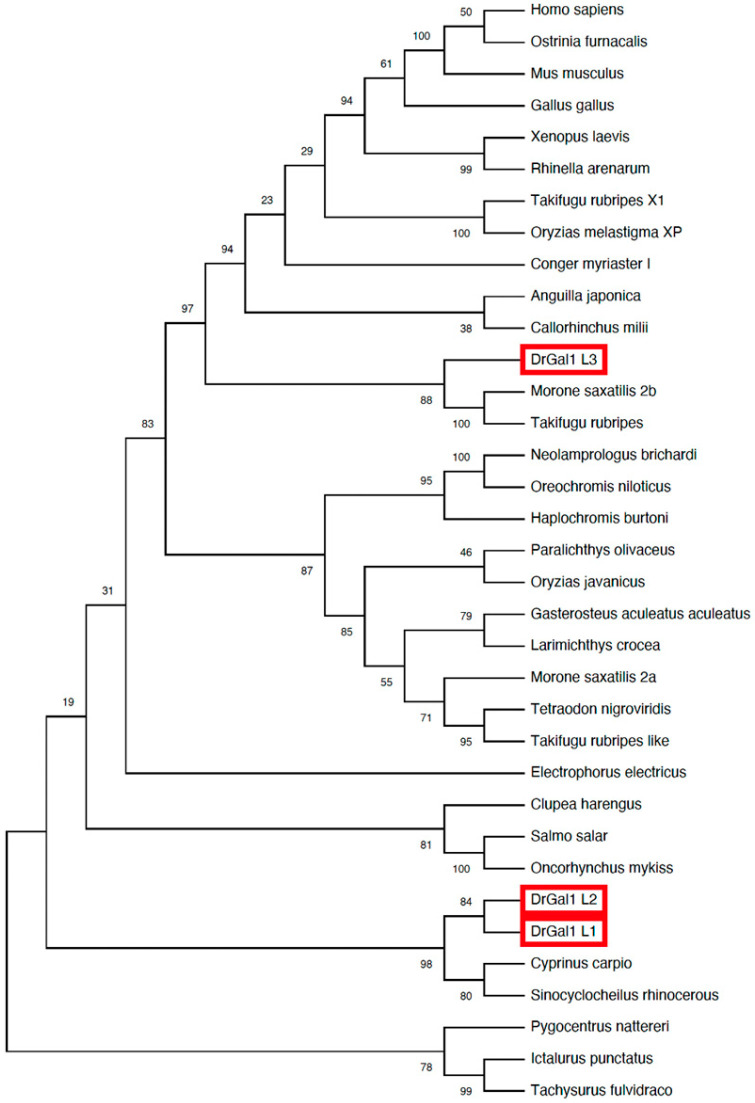
Phylogenetic analysis of Drgal1 isoforms. Phylogenetic tree of Drgal1 isoforms and other galectins from selected species. Analysis was performed using the ClustalW program, using the NJ (Neighbor Joining) method as described in Materials and Methods, and included galectin cDNA sequences from mostly prototype galectins from fish, as well as selected invertebrate, amphibian, bird, and mammalian species available in GenBank. Zebrafish Drgal1 isoforms -L1, -L2, and -L3 are boxed with red outlines.

The phylogenetic analysis of the Drgal1 isoforms (Figure 3) revealed a close relationship of the L1 and L2 isoforms with galectins from cyprinid species such as the common carp and the Chinese *Sinocyclocheilus* sp. In contrast, the L3 isoform was divergent, clustering with galectins from acanthuriformes, such as the striped bass (*Morone saxatilis*), and tetraodontiformes, such as the Japanese pufferfish (*Takifugu rubripes*).

### 2.4. The Drgal1-L2 Isoform Is Expressed at Higher Levels in Skin and Gills

We examined the expression of the Drgal1 isoforms by qRT-PCR in selected tissues that included mucosal epithelia, such as skin, gill, and gut, which constitute the interface of the fish with the external environment. The isoform-specific oligonucleotide primer sets (Sigma-Aldrich) designed to match and amplify both the zebrafish and fathead minnow galectin gene sequences previously obtained in our laboratory [23] are listed in Table 1.

**Figure 4:**
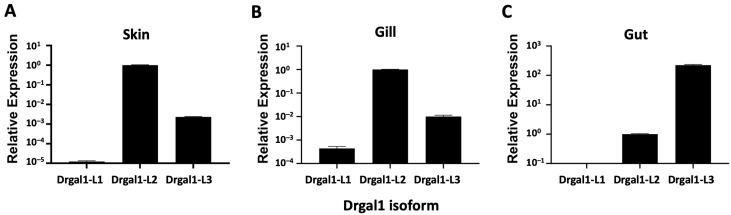
Tissue expression of Drgal1 isoforms. Tissue expression of Drgal1 isoforms was analyzed by qRT-PCR (SYBR green) as described in the Materials and Methods. Total RNA extracted from skin (**A**), gills (**B**), and gut (**C**) of adult wildtype zebrafish was reverse-transcribed to cDNA, then amplified using the real-time PCR method with gene-specific primers (Table 1). Data are representative of n = 3 from 2 independent experiments. The expression of Drgal1-L2 was normalized to 1.0 as the control across all tissues.

Results revealed that in healthy fish, the L2 isoform was preferentially expressed in skin and gills, whereas in the gut, the L3 isoform was prevalent over L2 (Figure 4). The L1 isoform was expressed the least in skin and gills and was virtually absent in the gut (Figure 4).

### 2.5. Drgal1-L2 Preferentially Binds -LacNAc

**Figure 5:**
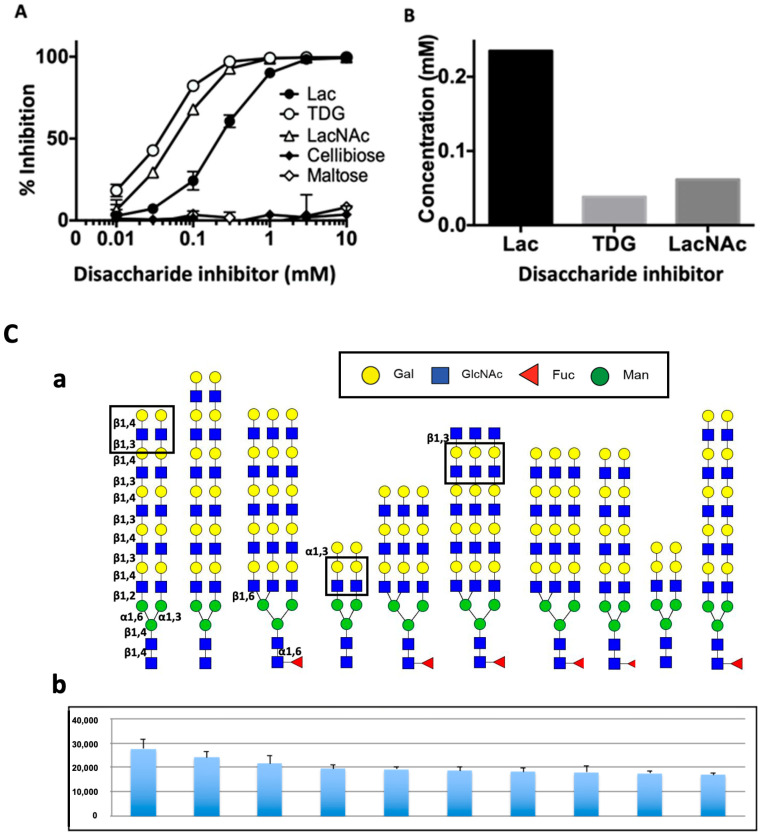
Drgal1-L2 preferentially binds to disaccharides LacNAc and TDG. (**A**) Characterization of Drgal1-L2 carbohydrate specificity by binding inhibition solid-phase assay: Drgal1-L2 binding to ASF was inhibited with increased concentrations of lactose (Lac), thiodigalactoside (TDG), LacNAc, cellobiose, or maltose, and the % inhibition was calculated relative to binding without any inhibitors. Inhibition curves of % inhibition vs. concentration (mM) of the test disaccharides are shown. Data are representative of n = 3 from 3 independent experiments. (**B**) Concentrations of disaccharide (mM) required for 50% inhibition are calculated and illustrated. (**C**) Glycan microarray analysis of Drgal1-L2 specificity: (**a**). Top binding ligands for Drgal1-L2 are shown. Both non-reducing terminal and internal LacNAc (Galβ1-4GlcNAc) moieties are boxed. (**b**). Drgal1-L2 binding to the microarray was tested at two concentrations as described in the Materials and Methods, and the binding profile of the 30 µg/mL sample is shown. Bars indicate the signal intensities of the ten top binding ligands illustrated above in (**a**).

Next, we investigated the carbohydrate specificity of Drgal1-L2 using solid-phase binding inhibition assay. Results showed that Drgal1-L2 preferentially binds LacNAc, followed by thiodigalactoside (TDG), a synthetic disaccharide that displays topological features similar to those of LacNAc, and weakly binds lactose (Figure 5A,B). Disaccharides cellobiose and maltose, known for lacking inhibitory capacity for galectins, failed to inhibit Drgal1-L2 binding, even at the highest concentrations. A glycan microarray analysis (Figure 5(Ca,Cb)) confirmed LacNAc (Galβ1-4GlcNAc) as the preferentially recognized disaccharide both as non-reducing terminal and internal moieties. The presence of a terminal Galα1-3 or GlcNAcβ1-3 linked to the subterminal LacNAc did not hinder recognition by Drgal1-L2.

### 2.6. Drgal1-L2 Preferentially Binds to Glycoproteins That Display LacNAc Moieties

**Figure 6:**
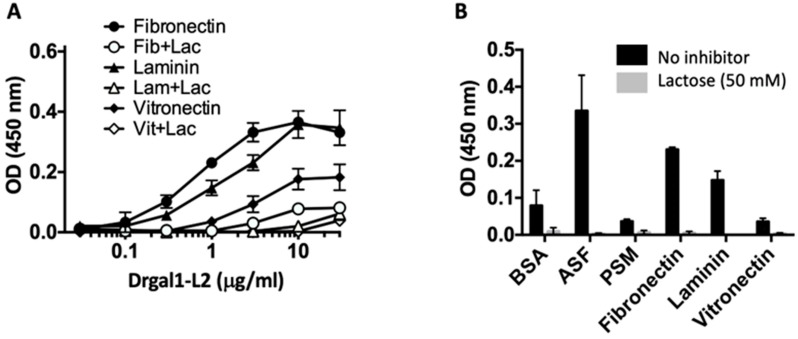
Binding of Drgal1-L2 to extracellular matrix glycoproteins. (**A**) Concentration-dependent binding of Drgal1-L2 to the extracellular matrix glycoproteins: the binding curves of increasing concentrations of Drgal1-L2 (0–30 µg/mL) in the absence or presence of 50 mM lactose (+Lac) to extracellular matrix glycoproteins fibronectin (Fib), laminin (Lam), or vitronectin (Vit) are shown. (**B**) The experiment was carried out as in “(**A**)” above at 1 µg/mL with additions of BSA, ASF and PSM as controls. Data are representative of n = 3 from independent experiments.

To examine the possibility that Drgal1-L2 could potentially recognize LacNAc moieties displayed by glycosylated macromolecules such as glycoproteins, we comparatively tested the binding of Drgal1-L2 to fibronectin, laminin, and vitronectin using asialofetuin, porcine stomach mucin, and BSA as controls. All three examined glycoproteins are not only present in the extracellular matrix but are also components of the epidermal mucus secretory system. Among the three tested glycoproteins, fibronectin behaved as the preferred ligand, followed by laminin and vitronectin, and the interactions were carbohydrate-specific, as Drgal1-L2 binding could be inhibited by lactose (Figure 6A). Like asialofetuin, fibronectin is rich in LacNAc moieties [30], to which Drgal1-L2 binds (Figure 6B). In contrast, PSM, which displays abundant blood-group A (GalNAc) and H (L-Fuc) moieties, was weakly bound by Drgal1-L2.

### 2.7. Drgal1-L2 Binds to Cell-Surface Receptors for Bacterial Adhesins

**Figure 7:**
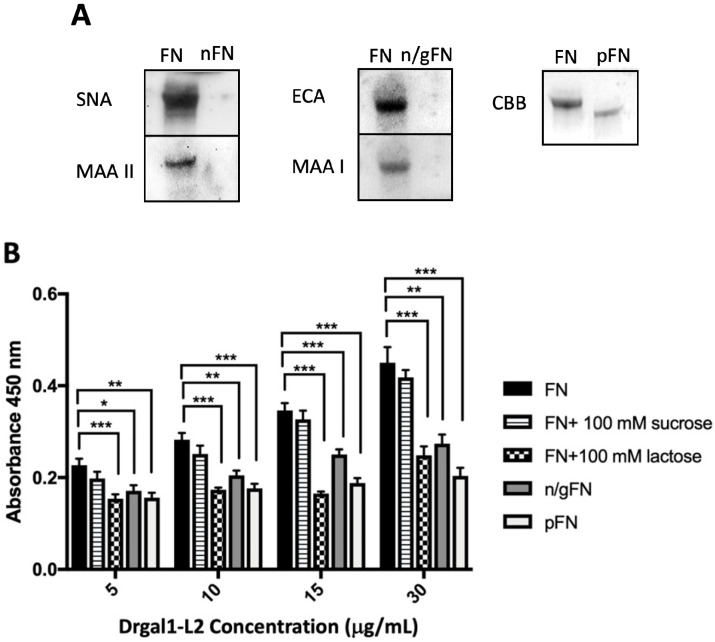
Binding of Drgal1-L2 to untreated and glycosidase-treated fibronectin. (**A**) Glycosylation of untreated and glycosidase-treated fibronectin (FN): The glycoprotein was treated with neuraminidase (nFN), followed by β-galactosidase (n/gFN), or PNGase F (pFN). The effectiveness of the treatments was analyzed by glycotyping with *Sambucus nigra* agglutinin (SNA; specific for Neu5Acα2-6Gal/GalNAc, tested at 5 mg/mL), *Maackia amurensis* II agglutinin (MAA II; Neu5Acα2-3Galβ1-3GalNAc, 5 mg/mL), *Erythrina cristagalli* agglutinin (ECA; Galβ1-4GlcNAc, 2.5 mg/mL), and *M. amurensis* I agglutinin (MAA I; Neu5Acα2-3Galβ1-4GlcNAc/Glc, 2.5 mg/mL). The pFN was analyzed according to its mobility on PAGE stained with Coommassie Brilliant Blue (CBB). (**B**) Deglycosylation of fibronectin reduces the binding of Drgal1-L2: The binding of Drgal1-L2 to untreated (FN) and glycosidase-treated fibronectin (n/g FN or pFN) is shown. Its binding to untreated FN in the presence of lactose or sucrose is included as a specificity control. Data are representative of n = 3 from 3 independent experiments. The average of replicate data (+/−) standard error of the mean standard deviation is represented. * *p* < 0.05, ** *p* < 0.005, and *** *p* < 0.0005 of experimental vs. control groups.

Fibronectin is ubiquitously expressed in zebrafish tissues and is homologous to the mammalian equivalent (Supplementary Figure S1). Given that fibronectin is not only a component of the extracellular matrix but it is also present on the cell surface and is considered the prototypical receptor for bacterial adhesins, we investigated its recognition and binding by Drgal1-L2 in further detail. To confirm that the binding of Drgal1-L2 to fibronectin observed in previous experiments (Figure 6A) is carbohydrate-dependent and specific, we treated fibronectin with glycohydrolases and examined Drgal1-L2 binding by solid-phase assays in the presence of specific sugar inhibitors. For this, commercial bovine fibronectin was treated with neuraminidase, followed by β-galactosidase, to cleave the terminal sialic acid and the subterminal galactose, respectively, or PNGase F to cleave N-linked glycans. The glycotyping and electrophoretic mobility results (Figure 7A) indicated that the glycohydrolase treatments were effective. The solid-phase assay for testing of the binding of Drgal1-L2 to the glycohydrolase-treated fibronectin indicated that the binding is carbohydrate-dependent and specific, as both the enzyme treatments and the presence of lactose reduced binding, whereas the addition of sucrose had no effect (Figure 7B).

**Figure 8:**
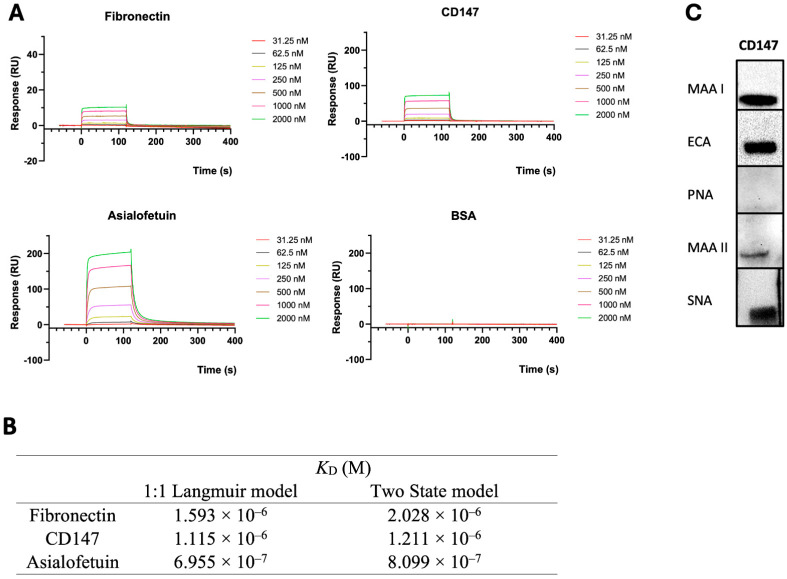
SPR analysis of interactions of Drgal1-L2 with cell-surface receptors for bacterial adhesins. (**A**) SPR analysis of Drgal1-L2 binding to fibronectin compared to other cell-surface receptors for bacterial adhesins (CD147) and positive and negative controls asialofetuin and BSA, respectively. Glycoproteins were immobilized on a CM5 sensor chip, and Drgal1 was run as analytes at 2× serial dilutions starting from 2000 nM to 31.25 nM. The sensorgram fitted using the 1:1 Langmuir model is shown. (**B**) Equilibrium dissociation constants (K_D_) from the experimental data fitted using two different models (1:1 Langmuir model and two-state model) with the Biacore T200 Evaluation Software (GE Healthcare). (**C**) Glycosylation of commercial CD147 was analyzed by glycotyping through Western blot with *Maackia amurensis* I agglutinin (MAA I; specific for Neu5Acα2-3Galβ1-4GlcNAc/Glc, tested at 2.5 mg/mL), *Erythrina cristagalli* agglutinin (ECA; Galβ1-4GlcNAc, 2.5 mg/mL), peanut agglutinin (PNA; Galβ1-3GalNAc, 5 mg/mL), *M. amurensis* II agglutinin (MAA II; Neu5Acα2-3Galβ1-3GalNAc, 5 mg/mL), and *Sambucus nigra* agglutinin (SNA; Neu5Acα2-6Gal/GalNAc, 5 mg/mL). Data are representative of n = 2 from 1 experiment.

Next, given that, in addition to fibronectin, the cell surface displays a variety of glycoproteins that could be recognized by Drgal1-L2, the binding affinity Drgal1-L2 for fibronectin was compared to that for CD147, another well-characterized cell-surface receptor for bacterial adhesins. Like fibronectin, CD147 is also expressed in zebrafish tissues, is homologous to the mammalian equivalent (Supplementary Figure S2), and could also be targeted by bacteria for adhesion to the mucosal epithelia. For this, the binding of Drgal1-L2 for both purified glycoproteins, i.e., fibronectin and CD147, was quantitatively examined by surface plasmon resonance (SPR) analysis and compared to ASF and BSA as controls (Figure 8A). To obtain the equilibrium dissociation constant (K_D_), the experimental data were fitted using two different models (the 1:1 Langmuir model and the two-state model) with Biacore T200 Evaluation Software (GE Healthcare) (Figure 8B). Sensorgrams fitted using the 1:1 Langmuir model are shown in the Figure 8A. Sensorgrams fitted using the two-state model are shown in Supplementary Figure S3. The K_D_ values obtained from the two models were comparable and showed consistent trends. Both sensorgrams fitted quality metrics (Rmax values and χ^2^ values) as low χ^2^ values, indicating good agreement between the experimental data and the fitted models. The estimated K_D_ values fall within the micromolar range, which is typical for many extracellular protein–ligand interactions [5]. Considering the estimated concentrations of Drgal1-L2 and its ligands in mucus or on epithelial surfaces, these affinities are compatible with physiologically relevant binding. Unlike the commercial bovine fibronectin used in this study, which is a natural product (glycosylation confirmed by results in Figure 7A), the CD147 tested in these experiments is a recombinant glycoprotein expressed in CHO cells. Therefore, we examined the glycosylation of recombinant CD147 by glycotyping with plant lectins of well-established specificity to verify that it was consistent with the natural equivalent, and the results confirmed its correct glycosylation (Figure 8C).

### 2.8. Drgal1-L2 Binds to Selected Glycans in Epidermal Mucus

**Figure 9:**
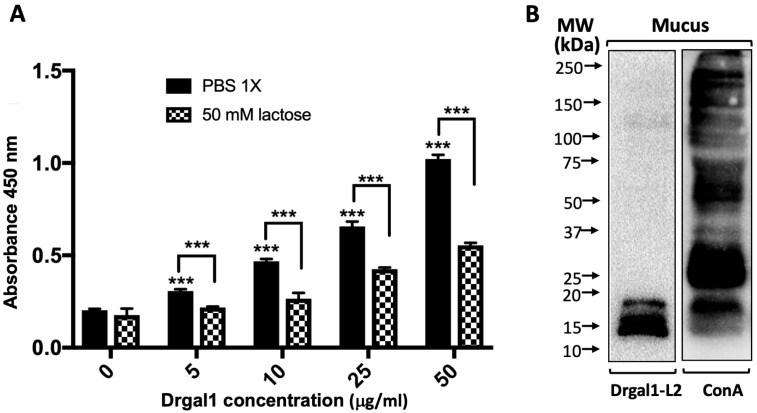
Drgal1-L2 binds to zebrafish epidermal mucus glycans. (**A**) The binding of increasing concentrations of biotinylated Drgal1-L2 (0–50 µg/mL) to 100 µg/mL zebrafish mucus with and without lactose analyzed by a solid-phase assay as described in the Materials and Methods is shown. The average of triplicate data (+/−) standard error of the mean (SEM) is represented. *** *p* < 0.0005 of 0 µg/mL vs. increasing concentrations and PBS 1X vs. lactose groups. (**B**) Samples (10 ug) of zebrafish mucus were run on SDS-PAGE gel, transferred to PVDF membranes, and overlaid with either 5 µg/mL of biotinylated Drgal1-L2 or 2.5 µg/mL of Concanavalin A (ConA). The blot was developed using chemiluminescence at 10 s exposure.

Next, we investigated the possibility that Drgal1-L2 secreted into the epidermal mucus could bind to mucus glycans and, perhaps, hinder the access of potentially infectious bacteria to the epithelial cell surface by cross-linking mucus components to bacterial surface glycans and immobilizing them within the mucus film. A solid-phase binding assay revealed that Drgal1-L2 binds to epidermal mucus in a dose-dependent and carbohydrate-specific manner, as lactose significantly inhibited binding (Figure 9A). A lectin overlay analysis of the electrophoretically fractionated epidermal mucus components showed that Drgal1-L2 binds to glycans with mobility corresponding to a 12–18 kDa range. The control consisting of a ConA overlay revealed the complexity of the mucus-glycosylated repertoire and confirmed the selectivity of Drgal1-L2 for relatively high-mobility components.

### 2.9. Drgal1-L2 Selectively and Specifically Binds to Bacterial Glycans

Binding of Drgal1-L2 to bacterial glycans was analyzed by a microbial glycan microarray, as described in the Materials and Methods. The galectin was selective for glycans from both Gram+ and Gram^−^ bacterial species, some of which are recognized fish pathogens (Table 2). The top binders were the OPS from *Providencia alcalifaciens*, several pneumococcal capsular polysaccharides, and *E. coli* OPS and LPS.

**Figure 10:**
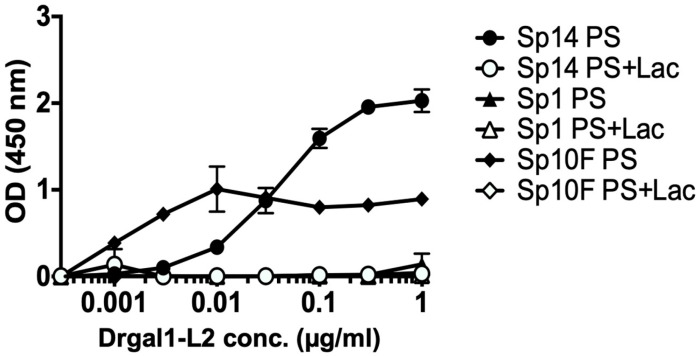
Binding of Drgal1-L2 to streptococcal exopolysaccharides. The binding of increasing concentrations of Drgal1-L2 to type 1 (Sp1 PS), type 10 (Sp10 PS), or type 14 (Sp14 PS) pneumococcal capsular polysaccharides in the absence or presence of 50 mM lactose (+Lac) analyzed by a solid-phase assay as described in the Materials and Methods. Data were collected from n = 3 from 2 independent experiments.

The specificity of the binding of Drgal1-L2 to streptococcal exopolysaccharides was analyzed in a solid-phase assay in which polysaccharides of three types (Type 1, Type 10, and Type 14) were immobilized on the wells (Figure 10). Results showed that Drgal1-L2 selectively binds to type 14 and 10 streptococcal exopolysaccharides in a dose–response and sugar-specific manner.

### 2.10. Drgal1-L2 Selectively and Specifically Binds to Whole Bacterial Cells

**Figure 11:**
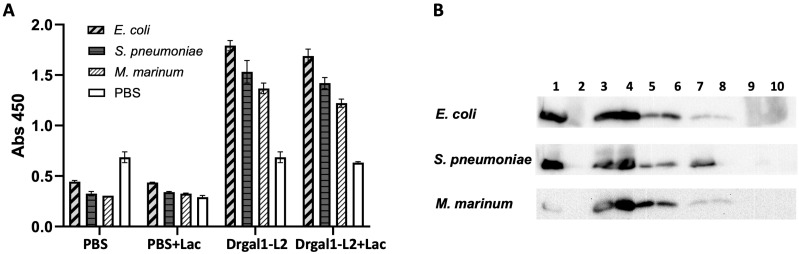
Binding of Drgal1-L2 to whole bacterial cells. (**A**) ELISA was performed to detect the binding of Drgal1-L2 to immobilized whole bacterial cells: Bacteria *E. coli*, *S. pneumoniae*, and *M. marinum* were immobilized onto the wells of a 96-well plate and incubated with PBS (as negative controls) or biotinylated Drgal1-L2 (10 µg/mL) with or without lactose (50 mM) as described in the Materials and Methods. Binding was detected with HRP-conjugated streptavidin. (**B**) Western blotting was carried out for the analysis of Drgal1-L2 adsorption on whole bacterial cells as described in the Material and Methods. The blot was developed using chemiluminescence at 10 s exposure. Lanes are as follows: 1. Drgal1-L2 input; 2. Bacteria input (untreated); 3. Drgal1-L2 (no lactose) unbound to bacteria; 4. Drgal1-L2 (pre-incubated with 100 mM lactose) unbound to bacteria; 5. wash from bacteria incubated with Drgal1-L2 (no lactose); 6. wash from bacteria incubated with Drgal1-L2 (with 100 mM lactose); 7. eluate with 500 mM lactose from bacteria incubated with Drgal1-L2 (no lactose); 8. eluate with 500 mM lactose from bacteria incubated with Drgal1-L2 (with 100 mM lactose); 9. bacteria incubated with Drgal1-L2 (no lactose) after elution with 500 mM lactose; 10. bacteria incubated with Drgal1-L2 (with 100 mM lactose) after elution with 500 mM lactose.

ELISA was performed to detect the binding of biotinylated Drgal1-L2 to immobilized whole bacterial cells (*E. coli*, *S. pneumoniae*, *M. marinum*) with or without lactose (50 mM) as described in the Materials and Methods. Results showed that Drgal1-L2 binds to all three bacterial species, with binding partially inhibited by lactose (Figure 11A). Western blotting was further carried out for the analysis of Drgal1-L2 adsorption on whole bacterial cells as described in the Material and Methods. Preliminary results showed that the binding of Drgal1-L2 to all three bacterial species (Figure 11B), especially *S. pneumoniae*, can be eluted by lactose (lane 7), suggesting a selective and carbohydrate-specific binding of Drgal1-L2 to the bacterial cells.

### 2.11. Drgal1-L2 Can Hinder the Adhesion of Bacteria to Epidermal Mucus

**Figure 12:**
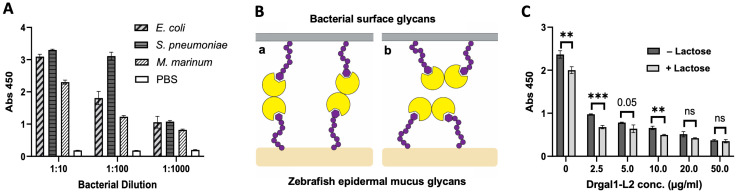
Potential role(s) of Drgal1-L2 in bacterial adhesion to zebrafish epidermal mucus. (**A**) Zebrafish epidermal mucus was immobilized on 96-well microtiter plates, blocked overnight with 3% BSA and incubated overnight at 4 °C with increasing dilutions (from 1:10 to 1:1000) of bacterial suspensions (*E. coli, S. pneumoniae,* and *M. marinum* or PBS as negative control), after which the supernatants were replaced with PBS and the bacterial adhesion to zebrafish mucus was assayed as described in the Materials and Methods. Data were collected from n = 3 from 1 experiment. (**B**) Schematic illustration of hypotheses about the potential role of Drgal1-L2 in bacterial adherence to epidermal mucus: Does Drgal1-L2 promote (“a”) or hinder (“b”) bacterial adhesion to epidermal mucus? (**C**) Zebrafish epidermal mucus was immobilized on 96-well microtiter plates, blocked overnight with 3% BSA, and incubated with increasing concentrations of rDrgal1-L2 (0–50 µg/mL) for 2 h at RT, followed by incubation with an *E. coli* suspension (1:100) in the presence or absence of lactose, and the bacterial adhesion to zebrafish mucus was assayed as described above. Data were collected from n = 3 from 1 experiment. The average of replicate data (+/−) standard error of the mean standard deviation is represented: ** *p* < 0.005, and *** *p* < 0.0005 of experimental vs. “+ Lactose” groups; ns: not significant.

As the first step of microbial infection of host tissues consists of adhesion of the bacteria to the external surface of the fish—either to the epidermal mucus film or directly to the skin that may have been depleted of mucus by mechanical trauma—we examined the possibility that Drgal1-L2 may hinder this process similarly to what we observed with IHNV [27]. For this, we first tested the adhesion of *E. coli*, *S. pneumoniae*, and *M. marinum* to zebrafish epidermal mucus in a solid-phase assay. Among the three abovementioned species, *E. coli* and *S. pneumoniae* were selected based on the results shown in Figure 10, in which they were identified by the microbial glycan microarray as strongly recognized by Drgal1-L2, whereas *M. marinum* was included as it is highly pathogenic for fish, including zebrafish. All three tested bacterial species adhered to the immobilized epidermal mucus (Figure 12A). Next, we examined the possibility that Drgal1-L2 may either promote (“a”) or hinder (“b”) bacterial adhesion to epidermal mucus (Figure 12B). For that, we pre-incubated the immobilized mucus with increasing concentrations of Drgal1-L2, removed the unbound galectin, and carried out the bacterial adhesion test. The results showed that Drgal1-L2 can partially inhibit adhesion of *E. coli* to zebrafish epidermal mucus in a dose-dependent manner, suggesting that model “b” is most likely to closely illustrate what takes place in the environmental setting (Figure 12C). The lactose control showed moderate albeit significant inhibitory activity with respect to bacterial adhesion in the absence of Drgal1-L2 pre-treatment, suggesting that either the bacteria bind to mucus glycans via galactosyl-binding adhesins or that endogenous galectins present in the immobilized mucus contribute to bacterial attachment by binding to the bacteria, which are inhibited by lactose.

## 3. Discussion

In this study, we characterized selected molecular and biochemical aspects of prototype galectin Drgal1 from zebrafish with the goal of gaining further insight into its potential role in innate immune functions against bacterial infection of mucosal epithelia. In a previous study, we demonstrated that Drgal1 can recognize the envelope glycoprotein of infectious hematopoietic necrosis virus (IHNV) and inhibit viral attachment to epithelial cells [27]. In a subsequent study, we determined the crystal structure of Drgal1 in complex with LacNAc and modeled the inhibitory effects of Drgal1 on the viral envelope spikes that would hinder IHNV attachment to the fish epithelial cell surface [28]. As a follow-up to these findings, in the present study, we first carried out the molecular and biochemical characterization of Drgal1, the tissue-specific expression of Drgal1 isoforms, and the identification of endogenous and exogenous ligands with the aim of addressing the question of whether Drgal1 present in epidermal mucus could similarly hinder the adhesion of bacteria to the fish epithelium to prevent infection. Our results revealed that Drgal1 comprises three isoforms—Drgal1-L1, -L2, and -L3, among which Drgal1-L1 and -L2 are closely related. All three isoforms are encoded by separate genes that lack a leader sequence and share all amino acid residues in the CRD involved in ligand recognition as described for most prototype galectins from mammals [3,5,8,11,29,31], as well as other fish [23,27,28,32,33,34] and amphibians [35,36,37,38,39]. The structural information of the CRD was supported by the glycan microarray studies and solid-phase binding and binding inhibition assays on Drgal1-L2 that confirmed that, like the mammalian equivalents, it preferentially binds to non-reducing terminal LacNAc (Galβ1,4GlcNAc) [2,5,11,29] but can also recognize internal LacNAc units.

Our results revealed that Drgal1 is abundant in skin, gills, and epidermal mucus. Among the three identified Drgal1 isoforms, Drgal1-L2 is highly expressed in the skin and gills and most likely comprises the majority of Drgal1 protein detected in the epidermal mucus. As the fish epithelial surfaces of skin, gills, and the gut constitute the interface of the animal with the external environment, most infectious challenges, whether viral, bacterial, or parasitic, start with attachment to the epithelial cell surface. These surfaces, however, display abundant mucus-producing cells (goblet, club, and sacciform cells) that secrete mucus as a continuous viscous film with high contents of soluble high molecular weight mucins that coat the mucosal epithelial surfaces [40,41,42]. Thus, the mucus layer functions both as a lubricant for the underlying mucosal epithelia and as a mechanical barrier that prevents potential pathogens from colonizing the epithelial surface of skin, gills, and the gut [42,43,44,45,46]. Furthermore, fish mucus contains multiple innate immunity factors, including lysozyme, proteases, antimicrobial peptides, and lectins, which function as a first barrier of defense against potential pathogens by immobilizing them, promoting their phagocytosis or directly killing them [47,48,49,50]. The antimicrobial role of epidermal mucus is further buttressed by the presence of immunoglobulins, complement proteins, and cytokines [49,50]. However, mucus secretion is significantly diminished in quantity/quality under the stressful environmental conditions of high-density aquaculture (e.g., temperature, pH, hypoxia, and pollutants). Additionally, loss of integrity of the mucus film as a result of mechanical trauma due to overcrowding enables pathogens to reach the epithelial surface and cause infection [51,52,53,54,55].

A wide variety of lectins have been identified in skin and epidermal mucus of both cartilaginous and bony fish. These include, for example, pentraxin in common skate (*Raja kenojei*) [56], mannose-binding C-type lectin in *Atlantic cod* [57], fructose-binding lectin from sea bass (*Dicentrarchus labrax*) [58], intelectin from catfish (*Silurus asotus*) [59], and galactose-binding C-type lectin from Atlantic salmon (*Salmo salar*) [60]. Among fish mucosal lectins, galectins have also been isolated and characterized from several fish species, including congerins I and II from the conger eel (*Conger myriaster*) [61,62,63], galectin AJL-1 from the Japanese eel (*Anguilla japonica*) [64,65], and galectin Msgal1 from the striped bass (*Morone saxatilis*) [32]. A comprehensive survey of 19 species and subspecies of freshwater eels (*Anguilla* spp.) suggested that galectins are virtually ubiquitous in their epidermal mucus [66]. The cell/gland source of mucus galectins, however, has been intriguing. While in the conger eel, galectins are secreted by club cells [63], in the striped bass, immunohistochemical analysis revealed that galectin Msgal1-L1 was absent in mucus-secreting cells but abundant in epithelial cells, as well as macrophages, suggesting that these cells are responsible for the release of galectin to mucus [32]. In mammals, prototype galectins such as Gal-1 are expressed by activated macrophages and secreted to the extracellular space with regulatory functions in innate and adaptive immunity [67,68]. In addition to regulatory functions, galectins released to the extracellular environment can also directly recognize and bind to potential pathogens and parasites [5,10,11,12,18,19,20]. Our observation that zebrafish galectin isoform Drgal1-L2 is expressed in mucosal epithelia and present in epidermal mucus is consistent with previous reports describing similar findings in other fish and vertebrates [32,69].

The finding that Drgal1-L2 showed preferential binding to LacNAc led us to examine the range of endogenous cell-surface glycan ligands and the exogenous bacterial moieties that display LacNAc moieties or topologically similar glycans that are recognized by the Drgal1 present in epidermal mucus, which would be consistent with a defense function at the interface of the animal with the external environment. Among the potential ligands for Drgal1 present at such an interface, endogenous glycans in epidermal mucus, such as soluble mucins, cell-surface and extracellular matrix glycoproteins or glycolipids, and glycans associated with the surface of potential pathogens, are likely targets. Our results showed that Drgal1-L2 binds to selected components of epidermal mucus—most likely, soluble mucins rich in O-linked oligosaccharides displaying LacNAc moieties, as observed for human Gal-1 and Gal-3, which bind to LacNAc moieties on N-linked and O-linked oligosaccharides of both soluble and cell-associated mucins [1,70,71,72].

Drgal1-L2 avidly recognizes glycoproteins displayed on the fish cell surface, such as fibronectin and CD147, both of which display LacNAc moieties [73,74,75]. Mammalian galectins have been reported to recognize several cell-surface glycans that display LacNAc moieties—namely, fibronectin; aβ-integrins; and transmembrane mucins such as MUC1, epidermal growth factor receptor, the Fas receptor, CD45, CD7, CD43, CD2, and many others [1,23,72,73,74,75,76,77,78,79]. Fibronectin is a glycoprotein present on the cell surface, extracellular matrix (ECM) and plasma that is involved in basic functions in cell adhesion, migration, growth and differentiation that are essential to early embryonic development, angiogenesis, and wound repair; thus, it is no surprise that it has been highly conserved throughout evolution of invertebrate and vertebrate lineages, from the earliest multicellular organisms (parazoa, e.g., sponges) to man [80,81,82,83,84,85,86,87]. The typical mammalian fibronectin structure consisting of the typical arrangements of domains formed by type I–III repeats, however, appears in the agnathans (jawless chordates such as lampreys and hagfish) and is expressed in all vertebrate taxa, with structural variability mostly limited to alternative splicing [82,83,86]. Fibronectin is highly N-glycosylated and displays LacNAc moieties [88] mostly as poly-N-acetyllactosamine chains, which are key to its multiple extracellular functions in cell adhesion and migration [89]. Given that LacNAc moieties are also the preferred ligands for prototype galectins [76], including Drgal1-L2 [23,90], the binding of galectins to fibronectins in various functional contexts has been widely reported [76]. The zebrafish expresses a unique truncated fibronectin, which is homologous to the mouse and mammalian equivalents, and shares over 50% of sequence identity and similar glycosylation [86].

Unlike fibronectin, which is loosely associated with the cell surface, CD147 (Cluster of differentiation 147), also known as EMMPRIN (Extracellular matrix metalloproteinase inducer) and Basigin, is a transmembrane signaling glycoprotein that belongs to the immunoglobulin superfamily [91,92,93,94]. Like fibronectin, however, CD147 is involved in basic functions in cell–cell interactions and differentiation associated with development, inflammation, tissue repair and remodeling and has been highly conserved in evolution, from protochordates (e.g., the lancelet *Amphioxus* spp.) to mammals, particularly in its transmembrane and cytoplasmic domains, the linker between the Ig-like domains, and its N-glycosylation sites [75,95,96,97,98,99,100]. Like fibronectin, CD147 is highly glycosylated, which is essential to its biological functions, and displays poly-N-acetyllactosamine moieties [74,101,102].

The strong binding of Drgal1-L2 to fibronectin and CD147 is particularly relevant, as these have been reported as important ligands for bacterial adhesins on the host cell surface [103,104,105,106]. Bacterial adhesins such as pili or fimbriae recognize and adhere to glycoprotein or glycolipid receptors on the host cell surface through protein–carbohydrate interactions [106,107,108,109,110,111]. In some examples, host cell-surface glycoproteins like fibronectin are not the actual receptors for the bacterial adhesin but it are “hijacked” to bridge the pathogen to the a5β1integrin receptor, and the resulting three-component complex (bacterium–fibronectin–integrin) facilitate the tight adherence of bacteria to host cells and promote the internalization of the bacteria into non-phagocytic host cells such as epithelial cells—a key step in bacterial infection and pathogenesis for many infectious diseases [105,106,107,108,109,110]. Some bacteria, such as *Staphylococcus aureus* and *S. pyogenes*, express fibronectin-binding proteins (FnBPs) or adhesins that specifically recognize and bind to fibronectin in the host cell surface and extracellular matrix (ECM) [108,109].

Several receptors for such bacterial adhesins have been identified in mammalian hosts, including humans, and in addition to integrins, they comprise immunoglobulin superfamily cell adhesion molecules (IgCAMs), cadherins, and extracellular matrix glycoproteins, such as collagen and fibronectin [110,111]. Bacterial adhesins commonly bind to the non-reducing terminal galactose or mannose in the covalently linked oligosaccharides of the receptors to initiate the infection process [112,113,114]. For example, while the FimH adhesin on type 1 pili of *E. coli* binds to mannosyl moieties on cell-surface integrins, adhesins from *Acinetobacter baumannii* and *Fusobacterium nucleatum* bind to galactosyl moieties on fibronectin [113,114].

In teleost fish, fibronectin has been identified as the receptor for bacterial adhesin MAM7, contributing to bacterial attachment to cells [115]. MAM7 is a multivalent adhesin found in many Gram-negative bacteria that helps them attach to host cells via fibronectin and phosphatidic acid on host cell membranes [115]. Several additional cell-surface receptors for bacterial adhesins have been identified in fish, including glycoproteins such as the scavenger receptor (SR) that interacts with the bacterial LPS and mediates internalization of the bacterial pathogen [116]. The *Salmonella* FimH adhesin specifically binds to calreticulin (CRT) and is responsible for bacterial adhesion and invasion [117]. The binding of Drgal1-L2 to endogenous “self” ligands such as mucus glycans and cell-surface glycoproteins is consistent with the established concepts about galectin recognition of ligands [118].

In addition to fibronectin, CD147 has also been reported as a cell-surface receptor for several bacterial adhesins, including the RadD adhesin from *Fusobacterium nucleatum*, which directly binds to the CD147 receptor on colorectal cancer cells, promoting bacterial attachment and potentially enhancing tumorigenesis [119]. Fimbrial adhesins PilE and PilV pili from *Neisseria meningitidis* also bind to CD147 as a receptor for adhesion to vascular endothelial cells; this binding has been identified as the initial step leading to septic shock [120]. Similarly, CD147 has been reported to promote infection by *Listeria monocytogenes* [121]. It is noteworthy that CD147 can also function as a receptor for various other pathogens and parasites, including *Plasmodium falciparum* and viruses like HIV-1 and SARS-CoV-2 [122].

The binding of Drgal1-L2 to exogenous “non-self” glycans on the bacterial surface such as LPS, OPS, and capsular exopolysaccharides that we observed in this study is consistent with recent findings of galectins recognizing various environmental bacteria, some of which are well-known fish pathogens, and suggests that Drgal1-L2 may function in defense against infection [9,11,12,14]. Based on the results from the glycan and microbial microarray analysis, interactions of Drgal1-L2 with bacteria would be mediated by recognition of glycans from Gram-negative and Gram-positive bacteria, such as LPS and capsular exopolysaccharides that display galactose moieties accessible to Drgal1-L2 binding. It is important to keep in mind that galectins not only are endowed with recognition properties for bacteria but can also display effector functions toward the recognized microbial pathogens, which facilitate their neutralization and clearance. For example, galectins-4 and -8 can bind to and kill *E. coli* that display human blood group B oligosaccharides on the surface by disruption of membrane integrity [12]. A similar bactericidal galectin-8 was identified and characterized from the tongue sole (*Cynoglossus semilaevis*) [14]. Furthermore, galectins can opsonize bacteria for phagocytosis and intracellular killing by oxidative stress [13,16,17]; therefore, it is also possible that, in addition to the galectin recognition properties, the effector activity of the mucus galectins described above could facilitate the defensive function of the epithelial macrophages.

Taken together, considering the range of “self” glycan ligands recognized by Drgal1-L2, such as mucus glycans and glycoprotein receptors fibronectin and CD147, and “non-self” glycans such as oligo- and polysaccharides on the bacterial surface, the information provided by this study enables the proposal of hypotheses about the potential defense mechanism(s) of Drgal1 against bacterial infection that are not mutually exclusive but likely synergic, as illustrated in Figure 12. One possibility is that the cross-linking of mucus glycans by Drgal1 can make the mucus film more viscous and less permissible of bacterial migration and hinder access to surface adhesin receptors needed for host entry and infection. The preliminary observation that Drgal1-L2 may hinder bacterial adhesion to the mucus film coating fish skin, gills and guts would further prevent bacterial adhesion to the fish external surface. For those bacteria that were able to enter the mucus film, the binding of Drgal1-L2 to both the bacteria and the mucus glycans could cross-link and immobilize the bacteria into the mucus matrix, which is periodically sloughed off, thereby clearing all potential pathogens able to adhere to the mucus film. Finally, in areas of mucosal epithelia in which the continuity of the mucus film has been disrupted due to mechanical abrasion or physiological stress, the binding of extracellular Drgal1-L2 to the bacterial adhesin receptors present on epithelial cells, such as fibronectin and CD147, would represent a backup strategy for hindering bacterial adhesion to the fish epithelial cell surface. The observations from this study must be interpreted with caution, however, as significant limitations include the use of an in vitro system with immortalized fish cell lines and the use of surrogate glycoproteins (fibronectin and CD147). These results will be corroborated by functional in vivo studies in zebrafish implementing genetic approaches—namely, gene silencing (e.g., siRNA) and editing (e.g., CRISPR) targeting both Drgal1-L2 and its epithelial surface glycoprotein ligands.

## 4. Materials and Methods

### 4.1. Reagents

Tricaine mesylate (MS-222), chloramphenicol, kanamycin, and β-mercaptoethanol (BME), neuraminidase, β-galactosidase were purchased from Sigma-Aldrich (St. Louis, MO, USA). PNGase F was purchased from New England Biolabs (Ipswich, MA, USA). The EZ-Link™Sulfo-NHS-SS-Biotin kit, Protein A-Sepharose, custom PCR primers, DAPI stain, Oligo (dT), RevertAid RT, RiboLock (RI), DreamTaq master mix, and dNTP mix were purchased from Thermo Fisher Scientific (Waltham, MA, USA). DNase, Nuclease free H_2_O, 10x cDNA mix, and EDTA were purchased from Invitrogen (Carlsbad, CA, USA). HRP-conjugated streptavidin and Alexa 488-conjugated streptavidin were purchased from Pierce (Waltham, MA, USA). The pET28b (+) vectors and Rosetta (DE3) pLysS competent cells, 25 U/mL Benzonase Nuclease, and lysozyme were obtained from Novagen (Madison, WI, USA). Cell culture media HBSS CaMg and L-15 Leibovitz were purchased from Gibco (Gaithersburg, MD, USA). The Trizol reagent was obtained from Fisher Scientific (Pittsburgh, PA, USA). SYBR Green ROX qPCR Master mix was purchased from Qiagen (Germantown, MD, USA). 1 X protease inhibitor cocktail set 1 was purchased from Calbiochem (San Diego, CA, USA). TMB substrate was purchased from SeraCare (Gaithersburg, MD, USA). Bovine serum fibronectin, human laminin and vitronectin, and anti-fibronectin antibody were purchased from Sigma Aldrich. Western Lightening Plus-ECL reagent, horseradish peroxidase (HRP), and carbohydrates used in binding inhibition assays were purchased from Sigma-Aldrich. Eight-chamber slides were purchased from Lab-Tek II (Santa Cruz, CA, USA). Purified capsular polysaccharides from *Streptococcus pneumoniae* were purchased from ATCC (Manassas, VA, USA). All other reagents were of the highest commercially available grade.

### 4.2. Animals, Collection of Epidermal Mucus and Tissues, and Preparation of Tissue Extracts

Zebrafish were raised and maintained at the Aquaculture Research Center at our institution (IMET) according to the standard previously described method [23,27]. Adult wildtype zebrafish (30–40 fish, 0.45–0.65 g) were euthanized with MS-222 (Sigma Aldrich) in dechlorinated tap water at a lethal concentration according to the protocol provided by the manufacturer. The fish were then placed in a Petri dish on ice. Mucus was collected from the dorsal and lateral surfaces of the fish by gently pressing a scalpel in a downward motion across the scales forward and back. Care was taken to not break the skin to avoid blood contamination of the mucus sample. Mucus from 5–10 fish was pooled to yield 200–500 µL of mucus with an average protein concentration of 5–8 mg/mL (1–2 mg/mL mucus/fish). Mucus pools were diluted in 50% volume of PBS 1X with Protease Inhibitor Cocktail Set I (PBS/PICSI; EMD Millipore Corp, Carlsbad, CA USA). Samples were vortexed vigorously for 3 × 30 s intervals and stored at −20 °C. After the mucus was collected, the fish were dissected to collect samples of the selected tissues (skin, gills, and gut) for Western blot and ELISA analyses. Each tissue sample was weighed and stored in a separate tube with an equal volume of PBS/PICSI, frozen at −80 °C, and crushed in a small mortar in an equal *w*/*v* of PBS 1X, followed by vortexing as above. The sample was then centrifuged at 10,000 rpm for 5 min, and the clear supernatant was transferred to a new tube and stored at −20 °C. Samples were quantified for protein concentration using a Bio-Rad Protein Assay Kit (Bio-Rad Laboratories, Hercules, CA, USA). For RNA extraction, skin, gill and gut samples were dissected from 15 healthy fish and pooled with the same tissues from 5 fish each in three pools. Total RNA was isolated using Trizol reagent following the manufacturer’s protocol and dissolved in 50 µL of sterile water. Samples were quantified for RNA concentration with a 260/280 ratio on a Nanodrop 2000 Spectrophotometer (Thermo Fisher Scientific).

### 4.3. Drgal1 Isoform Transcript and Gene Sequences

The gene sequences of Drgal1 isoforms (Drgal1-L1, Drgal1-L2, and Drgal1-L3) and their chromosomal location were obtained from GenBank. The transcript sequences for the Drgal1 isoforms were obtained by cloning or from GenBank as we previously reported [23].

### 4.4. Phylogenetic Analysis of Drgal1 Isoforms

Phylogenetic analysis of Drgal1 isoforms was performed using the ClustalW program, using the NJ (Neighbor Joining) method of Saitou and Nei [123] and included galectin genomic and cDNA sequences—mostly prototype galectins from fish—and selected invertebrate, amphibian, bird, and mammalian species available in GenBank to investigate evolutionary relationships and structural conservation.

### 4.5. Tissue Expression of Drgal1 Isoforms

The tissue expression of Drgal1 isoforms was analyzed by quantitative real-time PCR as previously described [27,124]. Briefly, total RNA from zebrafish tissues was treated with DNase I (Invitrogen) and reverse-transcribed into cDNA using the Reverse Transcriptase System (Promega, Madison, WI, USA) following the manufacturer’s protocol with oligo dT primers. For quantitative PCR, a 1 µL template of cDNA was mixed with 10 µL SYBR Green ROX qPCR Master mix (Qiagen), 8.2 µL of H_2_O and 0.4 µL of the oligonucleotide primers sets (Sigma-Aldrich) designed based on sequences of zebrafish Drgal1 isoforms Drgal1-L1, -L2, and -L3 [23,27] (Table 1). Amplification was carried out on a Fast 7500 Real-Time PCR System (Applied Biosystems, Waltham, MA, USA), with 40 cycles of 95 °C for 15 s, 62 °C for 1 min, and 70 °C for 1 s. Fluorescence measurements were taken at 70 °C for 1 s during each cycle. The relative expression level of each gene was calculated using the Livak (2^−ΔΔCt^) method after normalization to an endogenous β-actin control.

### 4.6. Expression and Purification of Recombinant Drgal1-L2

Expression and purification of recombinant Drgal1-L2 (rDrgal1-L2) were carried out as previously described [27,124]. Briefly, the histidine-tagged Drgal1-L2 (Genbank accession no. AY421704) in the pET28b (+) expression vector was transformed into *Escherichia coli* Rosetta^TM^ (DE3) pLysS competent cells (Novagen). Plasmid-carrying cells were grown in Luria–Bertani (LB) broth with antibiotics (Chloramphenicol, 34 mg/mL; kanamycin, 15 mg/mL), and recombinant protein expression was induced with 0.1 mM isopropyl D-thiogalactoside (IPTG) for 16 h at 23 °C with shaking. The soluble protein fraction was extracted from the cell pellet with BugBuster Protein Extraction Reagent (Novagen) containing 1 mM phenylmethylsulfonyl fluoride (PMSF), 1X Protease Inhibitor Cocktail Set I (Calbiochem), 20 mg/mL lysozyme, 25 U/mL Benzonase Nuclease (Novagen) and 0.07% β-mercaptoethanol (BME) and centrifuged at 14,000× *g* at 4 °C for 15 min. The clarified supernatant was loaded onto a pre-equilibrated chromatography column packed with 4 mL of divinyl sulfone-conjugated lactosyl-Sepharose slurry and washed with wash buffer (1:10 PBS, 0.07% BME); then, the purified rDrgal1-L2 was eluted with elution buffer (1:10 PBS, 0.07% BME, 100 mM lactose). Glycerol was added to a final concentration of 50% (*v*/*v*) and stored at −20 °C. In some experiments, rDrgal1-L2 was biotinylated as previously described [27,124].

### 4.7. Production of Anti-Drgal1 Antibodies

Anti-Drgal1 antiserum was prepared in New Zealand White rabbits at Duncroft (Lovetsville, FL, USA) by multiple subcutaneous and intramuscular injections of affinity-purified Drgal1 (100 µg/injection). Antiserum titers against Drgal1 were periodically monitored by enzyme-linked immunosorbent assay (ELISA) as previously described [23]. Immunoglobulins were purified on Protein A-Sepharose (Thermo Fisher Scientific) and confirmed for specificity in Western blot analyses by comparing side-by-side binding patterns to the affinity-purified Drgal1 and zebrafish crude tissue extracts as previously described [23]. Immunoglobulins from pre-immune serum (Day 0 of the immunization protocol) were purified following the same method [23]. Anti-Drgal1-L2 antiserum was prepared in New Zealand White rabbits at Duncroft, following a similar protocol as previously described [27]. Monitoring of the antiserum titers and specificity against rDrgal1-L2 and purification of immunoglobulins were carried out as reported elsewhere [23,27].

### 4.8. Whole-Mount Immunohistochemistry

Five days post fertilization (5 dpf), zebrafish embryos were fixed in freshly prepared 4% paraformaldehyde (PFA) solution for 10 min. After washing with PBS and blocking for 1 h with 3% BSA in PBS, the fixed embryos were incubated with the anti-Drgal1 antibody or immunoglobulins purified from pre-immune serum (1:100) overnight at 4 °C on a shaker. The next day, the embryos were washed and incubated with Cy3-conjugated goat anti-rabbit secondary antibodies (kindly provided by Dr. Adam Puche, University of Maryland School of Medicine, Baltimore, MD, USA) or Alexa 555-conjugated goat anti-rabbit antibody (Thermo Fisher Scientific). The embryos were counterstained with DAPI and mounted in ProLong Antifade mounting medium (Thermo Fisher Scientific). Images were captured on a confocal Leica SP8 TCS microscope with a 10× objective and 2.5× digital amplification with Fluoview V5.0.

### 4.9. Carbohydrate Specificity and Binding Affinity of Drgal1-L2

#### 4.9.1. Solid-Phase Binding Inhibition of Drgal1-L2 by Sugars and Glycoproteins

The carbohydrate specificity of Drgal1-L2 was determined by analyzing the binding of biotinylated rDrgal1-L2 to asialofetuin (ASF) in the presence of simple sugars or glycoproteins in a solid-phase assay as reported elsewhere [31,32,35]. Briefly, ASF (0.5 µg/100 µL/well) in 0.1 M Na_2_CO_3_/0.02% NaN3 (pH 9.6) was adsorbed onto the wells (37 °C, 3 h), and the bound glycoprotein was fixed with 2% formaldehyde (37 °C, 30 min). The plates were washed three times with PBS (azide-free) and incubated with the biotinylated rDrgal1-L2 conjugate (10 ng/100 µL/well for binding assays) or a pre-incubated mixture of an equal volume of Drgal1-L2 conjugate and varying concentrations of test carbohydrate ligands. After incubation (4 °C, 1 h), the plates were washed with ice-cold azide-free PBS–Tween 20 buffer, and binding was detected with HRP-conjugated streptavidin (Pierce) at a 1:1000 dilution. The plate was developed using TMB substrate, and the reaction was stopped by adding 1M HCl. Absorbance values were read at 450 nm on a SpectraMax340 Plate Reader (Molecular Devices, San Jose, CA, USA) controlled by SoftmaxPro software, version 1. The 50% inhibition concentrations (IC50) were calculated (Prism Version 6) as 50% binding activity relative to the control (100%; no inhibitor added). All experiments were carried out in triplicate and repeated at least twice.

#### 4.9.2. Glycotyping of Recombinant CD147

The glycosylation of the recombinant CD147 was visualized by the binding of labeled plant lectins of well-established specificity. For this, the lyophilized CD147 (50 µg) (expressed in CHO cells; Sino Biological, Paoli, PA, USA) was reconstituted in PBS pH 7.4 according to the manufacturer’s instructions for a final concentration of 200 µg/mL. The glycoprotein solution was mixed in loading buffer; heated; clarified by centrifugation; and resolved in 12-well, 4–15% gradient SDS-PAGE gels (Qiagen), then transferred to a PVFD membrane. The membranes were blocked with 3% BSA in PBS overnight, washed, and incubated with the biotinylated plant lectins (2.5–5.0 mg/mL) for 2 h at RT, followed by streptavidin–HRP for 1 h RT. The membranes were interrogated with the following lectins: *Maackia amurensis* I agglutinin (MAA I; specific for Neu5Acα2-3Galβ1-4GlcNAc/Glc, tested at 2.5 mg/mL), *Erythrina cristagalli* agglutinin (ECA; Galβ1-4GlcNAc, 2.5 mg/mL), peanut agglutinin (PNA; Galβ1-3GalNAc, 5 mg/mL), *M. amurensis* II agglutinin (MAA II; Neu5Acα2-3Galβ1-3GalNAc, 5 mg/mL), and *Sambucus nigra* agglutinin (SNA; Neu5Acα2-6Gal/GalNAc, 5 mg/mL). The membranes were washed and developed by chemiluminescence under 10 s exposure.

#### 4.9.3. Glycosidase Treatment of Glycoproteins

Bovine serum fibronectin (FN) (Sigma Aldrich) was treated with glycosidases to remove specific sugars. To cleave sialylated glycans bound to fibronectin, 500 µg of the glycoprotein was incubated with 3U of neuraminidase from *Clostridium perfringens* (Sigma Aldrich) overnight at 37 °C. The sample was then dialyzed in PBS 1X to remove cleaved sialic acid. This sample was designated as nFN. To cleave β-galactose from FN, 250 µg of nFN was incubated with 5U of β-galactosidase from *E. coli* (Sigma Aldrich) overnight at 37 °C. The sample was then dialyzed in PBS1X to remove cleaved galactose. The product was designated as n/gFN. To cleave N-linked glycans, 500 µg of FN was mixed with denaturing buffer (New England Bio Labs) and heated at 100 °C for 10 min, followed by the addition of G7 buffer (New England Bio Labs) and NP40 as recommended by the manufacturer, incubation with 3U of PNGase F (New England Bio Labs) overnight at 37 °C, and dialysis against PBS 1X to remove cleaved glycans. The product was designated as pFN. The effectiveness of the treatments was analyzed by glycotyping with SNA, MAA II, ECA, and MAA I at the concentrations indicated above. The effectiveness of the PNGase F treatment was analyzed by mobility on PAGE stained with Coomassie Brilliant Blue.

#### 4.9.4. Binding of Drgal1-L2 to Glycoproteins

Each well of a 96-well microtiter plate was coated with 100 ul of 2.5 µg/mL of either ASF, porcine stomach mucin (PSM), fibronectin (FN), laminin, vitronectin, n/gFN, or pFN and incubated at 37 °C for 2 h, blocked with 3% BSA, and incubated with increasing concentrations of biotinylated rDrgal1-L2 (0–30 µg/mL) in PBS 1X as described above. Specificity controls consisted of replacing rDrgal1-L2 with rDrgal1-L2 that had been pre-incubated with either lactose (50 or 100 mM), sucrose (100 mM), or PBS for 1 h at room temperature (RT). Binding was shown as absorbance values read at 450 nm.

#### 4.9.5. Glycan Microarray Analysis

Glycan microarray analysis following established procedures [125,126,127] was carried out at the National Center for Functional Glycomics (NCFG) at Beth Israel Deaconess Medical Center, Harvard Medical School, on version 5.0 of the CFG Glycan Microarray printed with 562 glycans in replicates of six (https://research.bidmc.org/ncfg/microarrays) accessed on 15 November 2025) The biotinylated rDrgal1-L2 (1 mg/mL) was dialyzed against PBS containing 10 mM β-mercaptoethanol (PBS/BME) and diluted with PBS/BME to 10 µg/mL or 30 µg/mL before being added onto the Glycam Microarray for analysis. The bound rDrgal1-L2 was detected with AlexaFluor 488 streptavidin conjugate (Invitrogen), and the signals from the two tested concentrations were examined for consistency. Experimental details of the protocol are available at https://www.functionalglycomics.org/protocol/cfgPTC_152 (accessed on 20 November 2025) for biotinylated galectins. The full datasets for the binding of Drgal1-L2 to the Glycan Microarray are available at https://www.functionalglycomics.org/glycan-array/1000056. (accessed on 25 November 2025) The results were analyzed by the GLycan Array Dashboard (GLAD) platform [128], and the glycan models were constructed using GlycoGlyph [129].

#### 4.9.6. Surface Plasmon Resonance (SPR) Analysis

SPR measurements were performed on a Biacore T200 instrument (GE Healthcare, Chicago, IL, USA) at 25 °C. Approximately 2000 resonance units (RU) of fibronectin, CD147, asialofetuin, and BSA were immobilized on a CM5 sensor chip in a sodium acetate buffer (50 μg/mL, pH 4.0) using the amine coupling kit provided by the manufacturer. A reference channel was immobilized with ethanolamine. Binding analyses were performed by injecting a solution of rDrgal1 over four cells at 2-fold increasing concentrations in HBS-P running buffer (10 mM HEPES, 150 mM NaCl, P20 surfactant 0.05% *v*/*v*, pH 7.4) containing 10 mM BME at a flow rate of 20 μL/min for 2 min and allowed to dissociate for another 5 min. The surface was regenerated after each cycle by injecting a 2 M MgCl_2_ water solution for 3 min at a flow rate of 30 μL/min. Kinetic analyses were performed by global fitting of the binding data to a 1:1 Langmuir binding model using BIAcore T200 evaluation v3.2 software.

#### 4.9.7. Binding of Drgal1-L2 to Zebrafish Epidermal Mucus

To examine the binding of Drgal1-L2 to zebrafish epidermal mucus, zebrafish epidermal mucus (100 µg/mL) was immobilized on a 96-well microtiter plate and blocked with 3% BSA overnight at 4 °C, followed by incubation with increasing concentrations of biotinylated rDrgal1-L2 (0–50 µg/mL) in PBS 1X, following a procedure similar to that for glycoproteins, as described above. The specificity control consisted of replacing rDrgal1-L2 with rDrgal1-L2 that had been pre-incubated with lactose (50 mM) for 1 h at RT. The recognition of specific components of zebrafish epidermal mucus by rDrgal1-L2 was examined by galectin overlays on PVDF membranes blotted with PAGE gels on which mucus had been resolved. For this, zebrafish mucus (5–10 µg/mL) was fractioned on 4–15% gradient SDS-PAGE Gel (SMOBio, Stellar Scientific, Owings Mills, MD, USA) and transferred to a PDFV membrane. The membranes were blocked overnight with 3% BSA at RT, then incubated with either 5 µg/mL of biotinylated rDrgal1-L2 or 2.5 µg/mL of biotinylated ConA (Vector Laboratories, Newark, CA, USA), followed by incubation with streptavidin-HRP. Detection was carried out by chemiluminescence using Western Lightning Plus-ECL reagent (PerkinElmer, Shelton, CT, USA) [130].

### 4.10. Interactions of Drgal1-L2 with Bacteria

#### 4.10.1. Bacterial Cultures

*Escherichia coli* was grown in LB (Difco) broth or agar (1.5% agarose) containing 10 µg/mL kanamycin and incubated at 37 °C overnight. *Streptococcus pneumoniae* was cultured as previously described [27,124]. Briefly, the bacterial culture suspension was streaked on agar plates containing 10% sheep blood and incubated overnight. Single bacterial colonies were inoculated in Todd–Hewitt broth and incubated at 37 °C with 5% CO_2_ overnight. *Mycobacterium marinum* (kindly provided by Dr. Russell Hill, Institute of Marine and Environmental Technology, Baltimore, MD, USA) was streaked on Middlebrook 7H10 agar plates with Dubos Oleic Albumin Complex. The bacterial streaks were inoculated in Middlebrook 7H9 broth and glycerol enriched with BSA, dextrose, and beef catalase and cultured in the dark at 30 °C for 72 h with continuous shaking [32]. Bacteria grown in liquid or agar plates were harvested by centrifugation at 4500 rpm for 15 min, resuspended in their specific liquid medium with 20% glycerol, and stored at −70 °C.

#### 4.10.2. Biotinylation of Bacteria

For biotinylation of bacteria, the bacterial pellets collected as described above were resuspended in biotinylation buffer (1 mM CaCl_2_, 0.5 mM MgCl_2_, 1.6 mM D-Biotin, pH 7.4), and sulfo-NHS-LC-Biotin was added to the suspension for a final concentration of 200 µM and incubated on ice for 30 min. The biotinylation reaction was quenched by adding TNKCM solution (50 mM Tris, 100 mM NaCl, 27 mM KCl, 1 mM CaCl_2_, 0.5 mM MgCl_2_; pH 7.4), followed by incubation at RT for 10 min. The bacteria were pelleted after centrifugation for 20 min at 4000× *g* at 4 °C, washed three times in TNKCM, and resuspended in 1 mL PBS (1×).

#### 4.10.3. Microbial Glycan Microarray

Analysis of the bacterial glycan recognition properties of Drgal1-L2 was carried out at the National Center for Functional Glycomics (NCFG) (https://research.bidmc.org/ncfg/microarrays) accessed on 3 December 2025) on version 4.2 of the Microbial Glycan Microarray (MGM) printed with about 400 glycans [e.g., capsular exopolysaccharides, O-antigen, lipopolysaccharide (LPS) derived from species/strains of Gram-positive and Gram-negative bacteria in replicates of six], following established procedures [131,132]. The biotinylated rDrgal1-L2 (1 mg/mL) was dialyzed against PBS containing 10 mM-ME (PBS/BME) and diluted with PBS/BME to 5 and 50 μg/mL before being added onto the MGM for analysis. The bound rDrgal1-L2 was detected with AlexaFluor 488 streptavidin conjugate (Invitrogen) as described above.

#### 4.10.4. Drgal1-L2 Binding to Streptococcal Polysaccharides

The binding of Drgal1-L2 to streptococcal exopolysaccharides (ATCC) was examined as described above for glycoproteins and epidermal mucus, with the required modifications. Briefly, the streptococcal exopolysaccharides were dissolved in sterile water at a concentration of 100 µg/mL, added to a 96-well plate, and air-dried overnight at 37 °C. The wells were then washed with PBS and blocked with 3% BSA, then incubated with various concentrations of biotinylated rDrgal1-L2 conjugate [pre-incubated with or without lactose (50 mM)]. Drgal1-L2 binding was detected as described above.

#### 4.10.5. Drgal1-L2 Binding to Whole Bacterial Cells

The binding of Drgal1-L2 to whole bacterial cells was examined using an ELISA-based assay as described previously [32]. Briefly, suspensions of *E. coli*, *S. pneumoniae*, and *M. marinum* were diluted and dispensed into EIA plates at 200 µL per well, incubated overnight at 4 °C, fixed with 1% PFA, and neutralized twice with 0.1% glycine for 1 h, then blocked with 3% BSA for 2 h at RT, followed by incubation with biotinylated rDrgal1-L2 (5 µg/mL) (pre-incubated with or without lactose 400 mM) for 1 h at RT, then with streptavidin-HRP, and development with HRP substrate. Binding signals from duplicate wells and from two independent experiments were recorded in a plate reader at 450 nm. The binding of Drgal1-L2 to whole bacterial cells was also examined by adsorption of rDrgal1-L2 on the bacterial surface and elution by competitive inhibition. For this, bacteria stored at −70 °C were centrifuged at 300 rpm for 10 min; the bacterial pellets were resuspended in PBS to 0.5 OD_550_, fixed overnight with 0.05% formaldehyde, washed thoroughly, and resuspended in PBS to 0.5 OD_550_. The bacterial suspensions in PBS were mixed with rDrgal1-L2 (100 µg/mL) and incubated for 2 h at RT, followed by centrifugation (4000× *g*), and washed three times with PBS. Controls were bacterial suspensions exposed to rDrgal1-L2 that had been pre-incubated with lactose (100 mM). Elution of the bacteria-bound rDrgal1-L2 was carried out by resuspending the washed bacterial pellets in PBS containing lactose (500 mM) and incubating the mixtures for 2 h at RT, followed by centrifugation (4000× *g* at 4 °C). The clear eluates and bacterial pellets were analyzed by Western blot as reported previously [130], first by resolving them on 10% SDS-PAGE gel, followed by transfer to a PVDF membrane. The membranes were blocked with 5% non-fat dry milk for 1 h at RT, then incubated with a 1:1000 dilution of purified anti-Drgal1-L2 antibody overnight at 4 °C, followed by incubation with a 1:1000 dilution of HRP-conjugated anti-rabbit IgG. Detection was carried out by chemiluminescence using Western Lightning Plus-ECL reagent (Perkin Elmer).

#### 4.10.6. Bacterial Adhesion Inhibition Assay

Adhesion of bacteria to epidermal mucus was examined by immobilizing zebrafish epidermal mucus on 96-well microtiter plates, including their blocking with 3% BSA overnight at 4 °C, followed by the addition of the biotinylated bacterial suspensions at increasing dilutions in PBS (from 1:10 to 1:1000); incubation overnight at 4 °C, followed by removal of the supernatants containing the non-adhered bacteria; and their replacement with PBS. Bacterial adhesion to the immobilized mucus was detected with HRP–streptavidin conjugate. The controls consisted of either the addition of lactose (100 mM) to the immobilized mucus incubated with the bacteria overnight at 4 °C or replacement of the zebrafish epidermal mucus with 3% BSA. To investigate the potential hindering of bacterial adhesion by rDrgal1-L2, 200 µL of rDrgal1-L2 at increasing concentrations (0–50 µg/mL) was added to the mucus-coated/BSA-blocked plates, followed by incubation for 2 h at RT, after which the biotinylated bacterial suspension (*E. coli* 1:100) was added and incubated as described above and the supernatants were replaced by PBS. The controls consisted of the addition of lactose (100 mM) as described above.

## 5. Conclusions

In this study, we characterized, in detail, the molecular and biochemical features of isoforms of zebrafish prototype galectin Drgal1 and their tissue-specific expression and localization. The Drgal1-L2 isoform showed a tissue distribution that is compatible with immune function(s) in mucosal tissues, and both the endogenous and exogenous microbial glycans that were identified as ligands recognized by the extracellular Drgal1-L2 were consistent with defense against bacterial infection at the interface of the animal with the external environment. Results revealed that Drgal1-L2 can bind to both bacterial and epidermal mucus glycans, as well as to fish epithelial cell-surface glycoproteins such as fibronectin and CD147, which have been shown to function as receptors for bacterial adhesins. The range of carbohydrate moieties recognized by Drgal1-L2 on the zebrafish cell surface, epidermal mucus, and bacterial glycans enabled the proposal of hypotheses about the potential mechanism(s) involved in its innate immune function(s) to be tested through ongoing experimental studies implementing gene silencing and editing approaches.

## Figures and Tables

**Table 1:** Primers used for the amplification of Drgal1 isoforms.

Primer	Sequence
Drgal1-L1 forward	5′-CGCGGAATGTTCGTGATG-3′
Drgal1-L1 reverse	5′-CCCTTGGATCCTAGCTTGGC-3′
Drgal1-L2 forward	5′-CCAGTGCACTATAGTGTGCAATTC-3′
Drgal1-L2 reverse	5′-TCATTGGTGAATGTGATTTTTATCT-3′
Drgal1-L3 forward	5′-GCAGCTCCACCAACAACTCAG-3′
Drgal1-L3 reverse	5′-CGTGTGTGAAGGCATCGTCT-3′

**Table 2:** Binding of rDrgal1-L2 to bacterial glycans in a microbial glycan microarray.

Chart #	BPS #	Bacteria/Strain	Name/Structure	Average	St. Dev.	%QC
132	140	*Providencia alcalifaciens* O5	OPS	40,781	4878	12
234	243	*Streptococcus pneumoniae* type 14 (Danish type 14)	197-X//Capsular PS	31,200	6944	22
244	253	*Streptococcus pneumoniae* type 54 (Danish type 15B)	241-X//Capsular PS	23,595	5730	24
236	245	*Streptococcus pneumoniae* type 19 (Danish type 19F)	205-X//Capsular PS	14,799	13,529	91
242	251	*Streptococcus pneumoniae* type 43 (Danish type 11A)	233-X//Capsular PS	6600	4850	73
134	142	*Providencia alcalifaciens* O19	OPS	6273	6646	106
31	36	*Escherichia coli* O40	OPS	4423	3045	69
237	246	*Streptococcus pneumoniae* type 20 (Danish type 20)	209-X//Capsular PS	2295	2100	92
224	233	*Escherichia coli* O55:B5 LPS- solution at 1 mg/mL	L5418-2ML (LPS) (Sigma)	2143	1033	48

## Data Availability

Datasets resulting from this study are stored in the authors’ laboratory and institutional servers. Glycan and Microbial Microarray datasets are available at the National Center for Functional Glycomics, Beth Israel Deaconess Medical Center, Harvard Medical School.

## Supplementary Materials

The following supporting information can be downloaded at: https://www.mdpi.com/article/10.3390/ijms27093827/s1.

## Abbreviations

CRD	Carbohydrate recognition domain
Drgal1-L2	*Danio rerio* galectin 1-like isoform 2
rDrgal1-L2	Recombinant Drgal1-L2
RT	Room temperature
ASF	Asialofetuin
PSM	Porcine stomach mucin
BSA	Bovine serum albumin
FN	Fibronectin
Sp1 PS	*Streptococcus pneumoniae* capsular polysaccharide type 1
Sp10 PS	*Streptococcus pneumoniae* capsular polysaccharide type 10
Sp14 PS	*Streptococcus pneumoniae* capsular polysaccharide type 14
HRP	Horseradish peroxidase
ABTS	Diammonium 2,2’-azinobis(3-ethylbenzothiazoline-6-sulfonate)
BME	2(β)-mercaptoethanol
PBS	Phosphate-buffered saline (0.01 M Na_2_HPO_4_/0.15 M NaCl/0.01% NaN_3_, pH 7.5)
PBS/BME	PBS containing 0.01 M BME
PBS (1:10)	PBS diluted 10-fold with water
SDS	Sodium dodecyl sulfate
PAGE	Polyacrylamide gel electrophoresis
PCR	Polymerase chain reaction
ORF	Open reading frame
Tris	Tris(hydroxymethyl) aminomethane
LPS	Lipopolysaccharide
OPS	O-antigen polysaccharide
PFA	Paraformaldehyde
ConA	Concanavalin A
MAA I	*Maackia amurensis* agglutinin I
ECA	*Erythrina cristagalli* agglutinin
PNA	Peanut agglutinin
MAA II	*Maackia amurensis* agglutinin II
SNA	*Sambucus nigra* agglutinin
NCFG	National Center for Functional Glycomics
