# Supplementary material for: A Zebrafish Galectin-1 Isoform Is Expressed in Skin and Gills and Binds to Bacteria, Bacterial Adhesin Receptors, and Epidermal Mucus Glycans
Source: Int J Mol Sci. 2026 Apr 25;27(9):3827. doi: 10.3390/ijms27093827 (PMC13163536; doi:10.3390/ijms27093827)
Supplement: Supplementary file 1 [file ijms-27-03827-s001.zip › ijms-4157841-supplementary.pdf]

International Journal of *Molecular Sciences*

MDPI

Article

A Zebrafish Galectin-1 Isoform Is Expressed in Skin and Gills and Binds to Bacteria, Bacterial Adhesin Receptors, and Epidermal Mucus Glycans

Chiguang Feng [1,†,‡], Kelsey Abernathy [1,†,§], Sheng Wang [1,2], Guanghui Zong [3], Nilli Zmora [4], Allison Shupp [1,5], Muddassar Iqbal [1], Lai-Xi Wang [3] and Gerardo R. Vasta [1,*]

1 Department of Microbiology and Immunology, University of Maryland School of Medicine, UMB, and Institute of Marine and Environmental Technology, Baltimore, MD 21202, USA
2 State Key Laboratory for Biocontrol, School of Life Sciences, Sun Yat-sen University, Guangzhou 510275, China
3 Department of Chemistry and Biochemistry, University of Maryland, College Park, MD 20742, USA
4 Department of Marine Biotechnology, Institute of Marine and Environmental Technology, University of Maryland Baltimore County, Baltimore, MD 21202, USA
5 Northeastern University, Boston, MA 02115, USA
* Correspondence: gvasta@som.umaryland.edu
† These authors contributed equally to this work.
‡ Current address: Center for Scientific Review, National Institutes of Health, Bethesda, MD 20817, USA.
§ Current address: Early Charm Ventures, Baltimore, MD 21230 USA.

Supplemental Figure 1. Amino acid sequence alignment of fibronectin from bovine, rabbit, and zebrafish

```
CLUSTAL O(1.2.4) multiple sequence alignment


AAU14809.1                  MTRE---SVKRLLLLLCIGGSVHCMPQSAGKSKRQAQQQQIHLDTVYEEARSLAIHESGC      57
sp|P07589.4|FINC_BOVIN      MLGGPGPGLLLLLAVLSLGTAVPSA--GASKSRRQAQQIVQ-----PQSPLTVSQSKPGC      53
XP_017198575.1              MLGGPGPGLLL-LSVLCLGTAVPST--AASKSKRQAQQIVQ-----PQSPVAVSQSKPGC      52
                            *       .:   * :*.:* :* .   .*.**:*****         :.  :::  : **

AAU14809.1                  NDNGRVYRMNDEWERPYMDSTLKCTCEG-ASGVKCRSKPAAEESCYDKFNARSYRVGETY      116
sp|P07589.4|FINC_BOVIN      YDNGKHYQINQQWERTYLGSALVCTCYGGSRGFNCESKPEPEETCFDKYTGNTYRVGDTY      113
XP_017198575.1              YDNGKHYQINQQWERTYLGNALVCTCYGGSRGFNCESKPEPEETCFDKYTGNTYRVGDTY      112
                             ***: *::*::*** *:..:* *** * : *.:*.***  **:*:**:...:****:**

AAU14809.1                  ERPKDNMIWDCTCIGSGKGKISCTIANRCHEGGNSYRIGDTWTRPHDTGDYMLECVCLGN      176
sp|P07589.4|FINC_BOVIN      ERPKDSMIWDCTCIGAGRGRISCTIANRCHEGGQSYKIGDTWRRPHETGGYMLECVCLGN      173
XP_017198575.1              ERPKDSMIWDCTCIGAGRGRISCTIANRCHEGGQSYKIGDTWRRPHETGGYMLECVCLGN      172
                            *****.*********:*:*:*************:**:***** ***:**.**********

AAU14809.1                  GKGEWTCKPVAERCYDDSLGSSYVVGQTWQKPYQGWMIVDCTCLGEGNGRITCTSRNRCN      236
sp|P07589.4|FINC_BOVIN      GKGEWTCKPIAEKCFDQAAGTSYVVGETWEKPYQGWMMVDCTCLGEGSGRITCTSRNRCN      233
XP_017198575.1              GKGEWTCKPIAEKCFDHAAGTSYVVGETWEKPYQGWMMVDCTCLGEGSGRITCTSRNRCN      232
                            *********:**:*:*.: *:*****:**:*******:*********.************

AAU14809.1                  DQDTRTSYRIGETWSKIDSSGHTLQCLCTGNGRGEWKCDRHAASHVIPAIGTGSTVTHRV      296
sp|P07589.4|FINC_BOVIN      DQDTRTSYRIGDTWSKKDNRGNLLQCICTGNGRGEWKCERHTSLQTTS-AGSGS--FTDV      290
XP_017198575.1              DQDTRTSYRIGDTWSKKDNRGNLLQCICTGNGRGEWKCERHASLQTTS-TGSGP--FSDV      289
                            ***********:**** *. *: ***:***********:**:: :.    *:*      *

AAU14809.1                  TPVMNQVNVLNELIEEGNCKTDSGVSYYNNMSWIRTQGTKEMLCTCVGGGISCEEQDGQS      356
sp|P07589.4|FINC_BOVIN      RTAIYQPQPHPQPPPYGHCVTDSGVVYSVGMQWLKTQGNKQMLCTCLGNGVSCQET-AVT      349
XP_017198575.1              RAAVYQPQPHPQPAPYGHCVTDSGVVYSEGMQWLKTQGNKQMLCTCLGNGVSCQET-AVT      348
                              .: * :   :    *:* ***** *  .*.*::***.*:*****:*.*:**:*  . :

AAU14809.1                  QVYGGNSGGQPCVFPFVFSGNTHYSCISEGRSDGQLWCSTTSDYDSDGLYSFCTQKN-LL      415
sp|P07589.4|FINC_BOVIN      QTYGGNSNGEPCVLPFTYNGKTFYSCTTEGRQDGHLWCSTTSNYEQDQKYSFCTDHT-VL      408
XP_017198575.1              QTYGGNSNGEPCVLPFTYNGRTFYSCTTEGRQDGHLWCSTTSNYEQDQKYSFCTDHTAVL      408
                            *.*****.*:***:**.:.*.*.*** :***.**:*******:*:.*  *****::. :*

AAU14809.1                  VTTRGGNSNGALCQFPFKYNGRNYTDCTAEGRRDGMKWCGTTADNDREQKYGFCPMAAHE      475
sp|P07589.4|FINC_BOVIN      VQTRGGNSNGALCHFPFLYNNHNYTDCTSEGRRDNMKWCGTTQNYDADQKFGFCPMAAHE      468
XP_017198575.1              VQTRGGNSNGALCHFPFLYNNHNYTDCTSEGRRDNMKWCGTTQNYDADQKFGFCPMAAHE      468
                            * ***********:*** **.:******:*****.******* : * :**:*********
```

Supplemental Figure 1 (continued). Amino acid sequence alignment of fibronectin from bovine, rabbit, and zebrafish

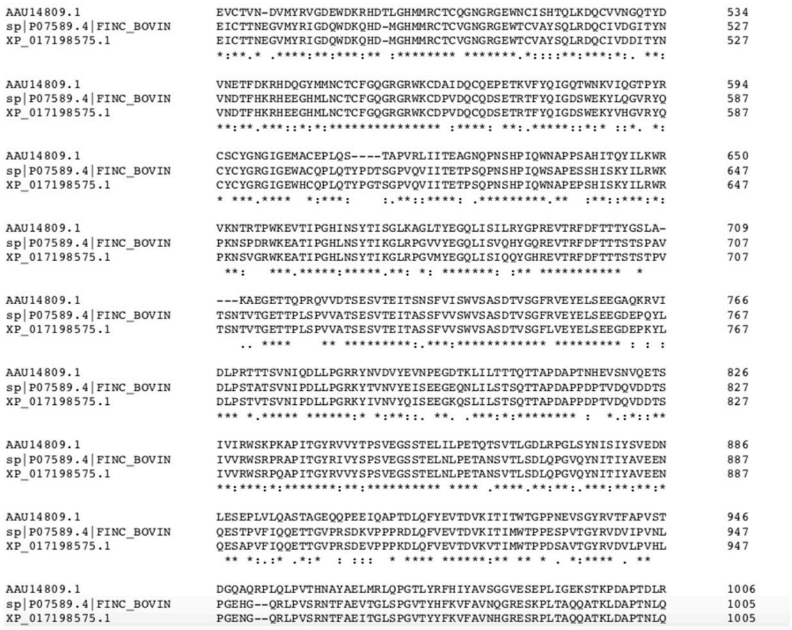

Supplemental Figure 1 (continued). Amino acid sequence alignment of fibronectin from bovine, rabbit, and zebrafish

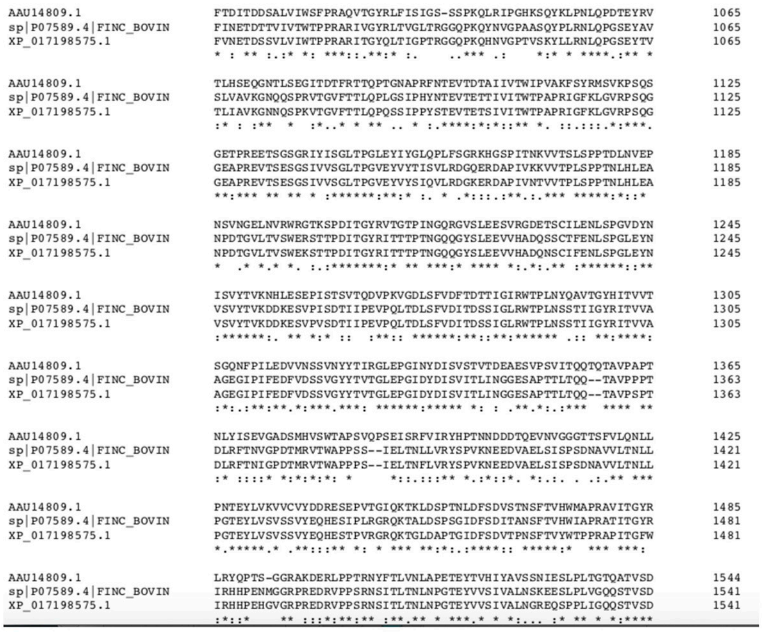

Figure S1. Amino acid sequence alignment of fibronectin from zebrafish, bovine, and rabbit: The aligned amino acid sequences of fibronectin from zebrafish (AAU14809.1), bovine (spP07589.4), and rabbit (XP_017198575.1) were obtained from the NCBI public database.

Supplemental Figure 2. Amino acid sequence alignment of CD147 from bovine, rabbit, and zebrafish

```
CLUSTAL O(1.2.4) multiple sequence alignment


XP_009294503.1      MEKKLFGAVLVICIGWFQAEAMTAGFIKSPLSQMKLIEDNAELDCEVIGNPIPEVQWWFI      60
BAC76828.1          ------------------------------------------------------------      0
ACD12686.1          ------------------------------------------------------------      0


XP_009294503.1      EGEEPDEIITQLFDGAREDRVHINATYIDHATSSLILTNLTFNDTGVYECRASNDPDRND      120
BAC76828.1          ------------------------------MAAALFVLLGFALLGTHGA--S-------      20
ACD12686.1          ------------------------------MA-----AVLFALLRAGGA--S-------      15
                                                    :      : *    .  .  *

XP_009294503.1      LRTPPKVKWIRSQANVIVFERATIMTSPEVNNATSATLSCNLTNPSSPIKGHYWVKNGKK      180
BAC76828.1          ------------GAAGTVF--TTVEDL-----GSKILLTCSLNDSATEVTGHRWLKGGVV      61
ACD12686.1          ------------AAAGTVT--TSVQSGDSWVQALALLLTCTLNTSAAGVTGHRWLKGKEV      61
                                 *   *     :::      .    *:*.*.  :: :.** *:*.

XP_009294503.1      IEESYSTNTDSYAEHFIEKADYHHSGIYTCVFLTEPQANATIEVKALPHIKASKSSENAN      240
BAC76828.1          LKEDALPGQK--TEFK--VDSDDQWGEYSCVFLPEPMGTANIQLHGPPRVKAVKSSEHIN      117
ACD12686.1          VKEDQLQGLH--TEHN--VTGDDRFGKYSCLFLPKDTGEATLTVDGPPRIKAVKKSEHAN      117
                    ::*.   . .  :*.     . .: * *:*:** :  . *.: :.. *::** *.**: *

XP_009294503.1      EKDKAVLSCGSQGYPSPTDWSWFKLPDAGERTPIFNGTDDKYEIKSTPNKTTLYVKDLDI      300
BAC76828.1          EGETAMLVCKSESVPPVTDWAWYKITDSEDK-ALMNGSESRFFVSSSQGRSELHIENLNM      176
ACD12686.1          EGDSVTLLCKSESFPSVTAWVWYKVADSGDQ-VIQNGSQSRFFISHSEAQSELHIKDLDL      176
                    * :.. * * *:. *  * * *:*: *: ::  : **::.:: :. :  :: *::::*::

XP_009294503.1      NEDMGFYQCQGTSEVGTGTDDIQLRVRSQLAALWPFLGIVAEVIILITIIFVYEKRRKPD      360
BAC76828.1          EADPGQYRCNGTSSKGSDQAIITLRVRSHLAALWPLLGIVAEVLVLVTIIFIYEKRRKPE      236
ACD12686.1          TSDPGEYACNGTSLQGTDTAVVTLRVRSRLAALWPFLGIVAEVLVLVTVIFIYEKRRKPD      236
                      * * * *:***  *:.   : *****:******:*******::*:*:**:*******:

XP_009294503.1      EIND----GSAPLKSNSATN--HKDKNVRQRNSN  388
BAC76828.1          DVLDDDDAGSAPLKSSGQHQN-DKGKNVRQRNSS  269
ACD12686.1          EVLDDEDAGAAPLKSSGHHVNDDKGKNVRQRNAS  270
                    :: *    *:*****..     .*.*******:.
```

Figure S2. Amino acid sequence alignment of CD147 (Basigin) from zebrafish, human, and rabbit: The aligned amino acid sequences of CD147 from zebrafish (XP_009294503.1), human (BAC76828.1), and rabbit (ACD12686.1) were obtained from the NCBI public database.

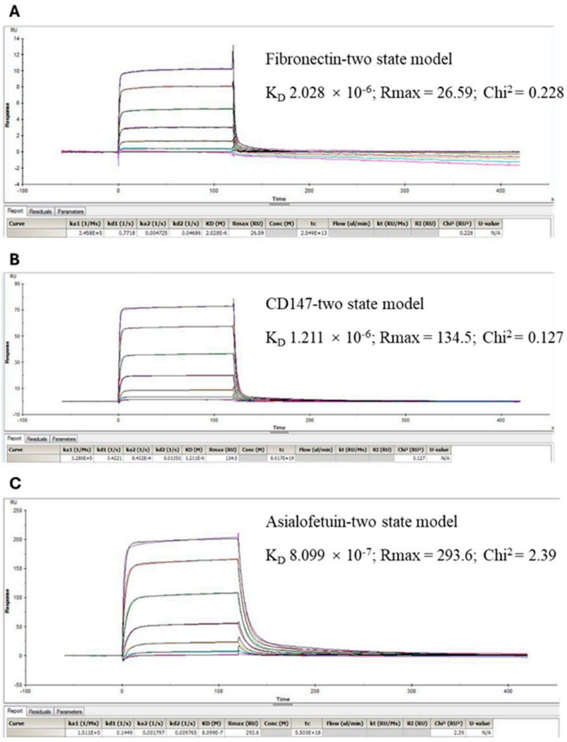

Figure S3. SPR sensorgrams fitted using the two-state model . The experimental data were fitted using two-state model.
